# Complex Bacterial Consortia Reprogram the Colitogenic Activity of *Enterococcus faecalis* in a Gnotobiotic Mouse Model of Chronic, Immune-Mediated Colitis

**DOI:** 10.3389/fimmu.2019.01420

**Published:** 2019-06-20

**Authors:** Isabella Lengfelder, Irina G. Sava, Jonathan J. Hansen, Karin Kleigrewe, Jeremy Herzog, Klaus Neuhaus, Thomas Hofmann, R. Balfour Sartor, Dirk Haller

**Affiliations:** ^1^Chair of Nutrition and Immunology, Technische Universität München, Freising, Germany; ^2^Division of Gastroenterology and Hepatology, University of North Carolina, Chapel Hill, NC, United States; ^3^Bavarian Center for Biomolecular Mass Spectrometry, Technische Universität München, Freising, Germany; ^4^ZIEL - Institute for Food & Health, Technische Universität München, Freising, Germany; ^5^ZIEL Core Facility Microbiome, Technische Universität München, Freising, Germany

**Keywords:** *Enterococcus faecalis*, ethanolamine utilization, gnotobiotic mouse models, microbial consortium, SIHUMI, IBD, IL-10 deficient mouse, RNA sequencing

## Abstract

Inflammatory bowel diseases (IBD) are associated with compositional and functional changes of the intestinal microbiota, but specific contributions of individual bacteria to chronic intestinal inflammation remain unclear. *Enterococcus faecalis* is a resident member of the human intestinal core microbiota that has been linked to the pathogenesis of IBD and induces chronic colitis in susceptible monoassociated IL-10-deficient (IL-10^−/−^) mice. In this study, we characterized the colitogenic activity of *E. faecalis* as part of a simplified human microbial consortium based on seven enteric bacterial strains (SIHUMI). RNA sequencing analysis of *E. faecalis* isolated from monoassociated wild type and IL-10^−/−^ mice identified 408 genes including 14 genes of the ethanolamine utilization (*eut*) locus that were significantly up-regulated in response to inflammation. Despite considerable up-regulation of *eut* genes, deletion of ethanolamine utilization (Δ*eutVW*) had no impact on *E. faecalis* colitogenic activity in monoassociated IL-10^−/−^ mice. However, replacement of the *E. faecalis* wild type bacteria by a Δ*eutVW* mutant in SIHUMI-colonized IL-10^−/−^ mice resulted in exacerbated colitis, suggesting protective functions of *E. faecalis* ethanolamine utilization in complex bacterial communities. To better understand *E. faecalis* gene response in the presence of other microbes, we purified wild type *E. faecalis* cells from the colon content of SIHUMI-colonized wild type and IL-10^−/−^ mice using immuno-magnetic separation and performed RNA sequencing. Transcriptional profiling revealed that the bacterial environment reprograms *E. faecalis* gene expression in response to inflammation, with the majority of differentially expressed genes not being shared between monocolonized and SIHUMI conditions. While in *E. faecalis* monoassociation a general bacterial stress response could be observed, expression of *E. faecalis* genes in SIHUMI-colonized mice was characterized by up-regulation of genes involved in growth and replication. Interestingly, in mice colonized with SIHUMI lacking *E. faecalis* enhanced inflammation was observed in comparison to SIHUMI-colonized mice, supporting the hypothesis that *E. faecalis* ethanolamine metabolism protects against colitis in complex consortia. In conclusion, this study demonstrates that complex bacterial consortia interactions reprogram the gene expression profile and colitogenic activity of the opportunistic pathogen *E. faecalis* toward a protective function.

## Introduction

Inflammatory bowel diseases (IBD) with the two dominant types Crohn's disease (CD) and ulcerative colitis (UC) are chronic relapsing inflammatory disorders affecting the distal intestine. Several factors appear to be involved in the pathogenesis of IBD, including genetic susceptibility (1, 2) together with diverse environmental triggers resulting in an inappropriate T-cell mediated activation of immunity toward components of the intestinal microbiota (3–5). IBD is associated with compositional (6–10) and functional (11, 12) changes of the intestinal microbiota referred to as dysbiosis. Dysbiotic changes associated with IBD are characterized by an overrepresentation of opportunistic pathogens and a loss of beneficial commensal organisms, indicating that the pathogenic potential of a dysbiotic community can be linked to certain organisms. Consequently, the specific contribution of individual bacteria to disease pathogenesis needs to be investigated in detail.

A relevant bacterial species in the context of IBD is *Enterococcus faecalis*. *E. faecalis* is a Gram-positive resident member of the human intestinal core microbiota harboring a number of pathogenic traits, which explains the association of this bacterium with inflammatory diseases and fatal nosocomial infections (13–15). Relative to healthy volunteers, the abundance of enterococci is increased in fecal samples of CD patients (16–18). This is in line with a high frequency of *E. faecalis* housekeeping and virulence genes in CD cohorts (19) and a high likelihood of the presence of virulence factors in *E. faecalis* isolates originating from inflamed IBD mucosa (20). UC patients have increased numbers of mucosa-associated *E. faecalis* correlating with disease activity (21) and a high *E. faecalis-*specific IgG sero-reactivity (22).

The dual characteristics of *E. faecalis* as core member of the human intestinal microbiota and as an opportunistic pathogen make this bacterium an ideal model to study microbe-host interactions relevant to the development of chronic colitis in a genetically susceptible host. In the *IL10*^*tm1Cgn*^ mouse (IL**-**10^−/−^), which is a well-characterized model of human chronic colitis, monoassociation with *E. faecalis* induces severe intestinal inflammation, whereas wild type mice remain disease-free (23–26). In previous studies, we could show that certain bacterial structures contribute to the colitogenic activity of *E. faecalis* in monoassociated IL-10^−/−^ mice (27, 28).

Using germ-free wild type and IL-10^−/−^ mice monoassociated with *E. faecalis* or colonized with a colitogenic human enteric bacterial consortium (SIHUMI) (29), we aim to unravel the functional relevance of *E. faecalis* in colitis development with regard to gene expression and interactions with co-colonizing bacteria. Based on RNA sequencing (RNA seq) analysis, we characterize the role of ethanolamine (EA) utilization for *E. faecalis* survival and colitogenic activity in a susceptible host.

The intestine is a rich source of the metabolite EA, which is derived from phospholipid phosphatidylethanolamine of bacterial and eukaryotic cell membranes (30, 31). Bacteria capable of EA catabolism can utilize this compound as a source of carbon and/or nitrogen to promote growth as well as a signal influencing virulence during host colonization (32). EA utilization is a well-recognized property of diverse pathogens, for example *Salmonella* and enterohemorrhagic *Escherichia coli* (EHEC) (33–37). The only commensals known to carry EA utilization genes are *E. faecalis* and some commensal strains of *E. coli* (32, 38–41). *E. faecalis* catabolizes EA using enzymes encoded by the *eut* genes that are contained in a locus consisting of 19 genes. The *eut* gene expression is controlled by the EutVW two-component system, comprising the sensor histidine kinase EutW and the response regulator EutV (42, 43). Using an *E. faecalis eutVW* double deletion mutant (43), we are able to establish a novel correlation between *E. faecalis* EA utilization and an attenuation of the pro-inflammatory host response in the presence of a complex bacterial consortium.

Using immuno-magnetic separation, we are able to purify wild type *E. faecalis* cells from the colon content of SIHUMI-colonized wild type and IL-10^−/−^ mice and analyze the gene expression in the presence of other bacteria. We show that *E. faecalis* adapts transcriptionally to the other co-colonizing bacteria. To unravel whether these transcriptional alterations have functional consequences, we colonized wild type and IL-10^−/−^ mice with the SIHUMI consortium in the presence and absence of *E. faecalis*. We determine a protective activity of *E. faecalis* in SIHUMI colonized susceptible mice, demonstrating that complex bacterial consortia interactions can reprogram the colitogenic activity of the opportunistic pathogen *E. faecalis* toward a protective function.

## Materials and Methods

### Bacterial Strains and Cultivation

For this study we used the characterized human oral *E. faecalis* strain OG1RF and an isogenic *eutVW* double deletion mutant kindly provided by Danielle Garsin (Department of Microbiology and Molecular Genetics, The University of Texas Health Science Center at Houston, Houston, Texas, USA) (43). *E. faecalis* strains (Table 1) were cultivated in Brain Heart Infusion (BHI) broth or on BHI agar (BD Difco) at 37°C under aerobic conditions. The SIHUMI (29) consortium (Table 1) was kindly provided by R. Balfour Sartor (Division of Gastroenterology and Hepatology, University of North Carolina, Chapel Hill, NC, USA). All SIHUMI strains were cultivated under anaerobic conditions on Wilkins-Chalgren-Agar (WCA, Thermo Fisher Scientific) or in WCA broth supplemented with 0.05% (w/v) L-cystein (Carl Roth) and 0.002% (w/v) dithiothreitol (Sigma Aldrich). Of note, a different strain set for rats is also called SIHUMI and should not be mistaken (46). For colonization of germ-free mice with the SIHUMI consortium, the bacterial mixture was prepared as follows. Strains were grown individually in Hungate tubes for 24 h. A mixture of all strains with an equal cell density of 1 × 10^9^ cells/ml was prepared in sterile glass tubes, mixed 1:1 with sterile glycerol in culture medium (40% v/v, gassed with N_2_) and sealed with a rubber septum. The bacterial mixture was stored at −80°C until use.

**Table 1:** Bacterial strains used in this study.

**Strain**	**Characteristics**	**References**
*Enterococcus faecalis* OG1RF	Wild type	(44)
*Enterococcus faecalis* Δ*eutVW*	OG1RF *eutV* and *eutW* double deletion mutant	(43)
*Escherichia coli* LF82	Wild type	(45)
*Ruminococcus gnavus* ATCC 29149	Wild type	(29)
*Bacteroides vulgatus* ATCC 8482	Wild type	(29)
*Faecalibacterium prausnitzii* A2-165	Wild type	(29)
*Lactobacillus plantarum* WCFS1	Wild type	(29)
*Bifidobacterium longum* subsp. *longum* ATCC 15707	Wild type	(29)

### Animal Experiments—Animal Housing

Colonization of germ-free 129S6/SvEv wild type and IL-10^−/−^ mice was performed in the National Gnotobiotic Rodent Research Center, University of North Carolina, USA and in the Core Facility Gnotobiology of the ZIEL—Institute for Food & Health, Technical University Munich, Germany. Mice were fed a standard chow diet (V1124-300, ssniff Spezialdiäten GmbH) *ad libitum*. The mice were kept in flexible film isolators ventilated via HEPA-filtered air at 22 ± 1°C with a 12-h light/dark cycle. Littermates were used when possible, or siblings from multiple breeders were combined and randomly assigned before assignment to experimental groups. The group size is a maximum of 5 mice per cage (floor area ~540 cm^2^). For enrichment the animals were provided with bedding, nesting material and an autoclavable cottage.

### Animal Experiments—Experimental Design

For monoassociation, germ-free 129S6/SvEv wild type and IL-10^−/−^ mice were colonized at the age of 10–12 weeks with either *E. faecalis* wild type OG1RF or the Δ*eutVW* mutant strain by oral and rectal swab. The mice were sacrificed after different periods of colonization (1, 4, 8, 12, 16, 20, and 24 weeks).

For colonization with the SIHUMI consortium, the bacterial mixture was prepared as described above. Germ-free 129S6/SvEv wild type and IL-10^−/−^ mice were colonized at the age 10 weeks with 1 × 10^8^ bacteria/strain by repetitive oral gavage (at week 0 and week 1). The composition of the bacterial consortia differed between the experimental groups as shown in Table 2. The mice were sacrificed after colonization periods of 4 or 16 weeks. A schematic overview of all experimental setups is included in the Supplementary Material.

**Table 2:** Composition of the bacterial consortium for the respective experimental groups.

**Experimental group**	**Strains included in consortium**
SIHUMI (29)	*E. coli, E. faecalis, R. gnavus, B. vulgatus, F. prausnitzii, L. plantarum, B. longum*
SIHUMI—*E. faecalis*	*E. coli, R. gnavus, B. vulgatus, F. prausnitzii, L. plantarum, B. longum*
SIHUMI *ΔeutVW*	*E. coli, E. faecalis ΔeutVW, R. gnavus, B. vulgatus, F. prausnitzii, L. plantarum, B. longum*

### Preparation of Bacterial Lysates

Bacterial lysates for stimulation of mesenteric lymph node (MLN) cell cultures were prepared as described previously (29). Details are provided in the Supplementary Material.

### Bacterial DNA Extraction

Bacterial cells were lysed by mechanical lysis using a FastPrep-24 (MP Biomedicals). DNA was isolated using phenol/chloroform/iso-amyl alcohol (Carl Roth) and contaminating RNA was digested with 0.1 μg/μl RNase A (VWR) for 30 min at 37°C. Subsequently, the DNA was purified using the gDNA clean-up kit (Macherey-Nagel) according to the manufacturer's instructions.

### Bacterial Community Analysis via Quantitative Real-Time Polymerase Chain Reaction (qPCR) Assay

For bacterial community analysis, oligonucleotide primers were used to target species-specific regions of the 16S ribosomal RNA (rRNA) gene. Quantification was performed using the LightCycler 480 Universal Probe Library System (UPL, Roche). Primers (Sigma Aldrich) and corresponding probes (Roche) are listed in Table 3. Positive and negative controls were included in every run. Standard curves were generated for each bacterial strain and used to enumerate the 16S rRNA gene copy numbers per species in intestinal content. 16S rRNA gene copy number per ng DNA was calculated via the molecular weight of the genome and 16S rRNA gene copy numbers/genome. The abundance of each bacterium was calculated as previously described (29) using the following formula: [(copies of 16S rRNA gene for a specific SIHUMI bacterium/cumulative copies of 16S rRNA gene of all bacteria) × 100].

**Table 3:** Species specific 16S rRNA gene-targeted primers and UPL probes used for bacterial community analysis.

**Target bacterial species**	**Oligonucleotide sequence (5'-3')**	**UPL probe**	**References**
*Escherichia coli* LF82	F: GGGACCTTAGGGCCTCTTG R: GCCTAGGTGAGCCTTTACCC	104	This study
*Enterococcus faecalis* OG1RF	F: GGTCATTGGAAACTGGGAGA R: TTCACCGCTACACATGGAAT	91	This study
*Ruminococcus gnavus* ATCC 29149	F: GGGACTGATTTGGAACTGT R: CGCATTTCACCGCTACACTA	55	This study
*Bacteroides vulgatus* ATCC 8482	F: CGCAACCCTTGTTGTCAGT R: CATCTTACGATGGCAGTCTTGT	73	This study
*Faecalibacterium prausnitzii* A2-165	F: TATTGCACAATGGGGGAAAC R: CAACAGGAGTTTACAATCCGAAG	69	This study
*Lactobacillus plantarum* WCFS1	F: CGAAGAAGTGCATCGGAAAC R: TCACCGCTACACATGGAGTT	35	This study
*Bifidobacterium longum* subsp. *longum* ATCC 15707	F: TGGTAGTCCACGCCGTAAA R: TAGCTCCGACACGGAACC	42	This study
Total bacteria	F: YAACGAGCGCAACCC R: AAGGGSCATGATGAYTTGACG	69	(47)

### Isolation of *E. faecalis* Cells From Intestinal Content by Immuno-Magnetic Separation

The isolation method was modified according to protocols of Nolle (48). Frozen colonic content from SIHUMI-colonized mice was disrupted by bead beating in 1 ml RNAlater (Sigma Aldrich) using glass beads (1 mm, Carl Roth). Subsequently, samples were diluted with wash buffer (PBS, 0.5% biotin-free BSA, 10% RNAlater) and filtered through three cell strainers with decreasing pore size (VWR; 100, 70, and 30 μm, respectively). The samples were kept on ice throughout and only pre-chilled buffer solutions were used. The bacterial cells were pelleted by centrifugation and resuspended in 1 ml wash buffer. The samples were mixed with 10 μl *Enterococcus*-specific antibody (biotinylated, Thermo Fisher Scientific PA1-73123) and incubated for 10 min at 4°C under gentle agitation. The samples were centrifuged for 2 min at 9600 × g. After a washing step, the cells were resuspended in 100 μl wash buffer. Ten μl Streptavidin MicroBeads (Miltenyi Biotec) were added per sample. After incubation for 15 min at 4°C under gentle agitation, the cells were washed with wash buffer and resuspended in 500 μl separation buffer (PBS, 0.5% biotin-free BSA, 2 mM EDTA, 10% RNAlater). Magnetically labeled *E. faecalis* cells were isolated using MACS filter columns (LS, Miltenyi Biotec) in a MidiMACS Separator (Miltenyi Biotec) according to the manufacturer's instructions and total bacterial RNA was isolated.

### Bacterial RNA Isolation

Bacterial total RNA was isolated as described previously (49). Details are provided in the Supplementary Material.

### Microbial Total RNA Sequencing (RNA Seq)

#### RNA Seq of *E. faecalis* Isolated From Monoassociated Mice

Bacterial RNA prepared from the colonic content of *E. faecalis* OG1RF monoassociated wild type and IL-10^−/−^ mice (*n* = 8 mice/group; colonization period: 16 weeks) was sequenced as described previously (28). RNA isolation and rRNA depletion was done by Jonathan Hansen and Sandrine Tchaptchet from the Division of Gastroenterology and Hepatology of the University of North Carolina in Chapel Hill. Library preparation and sequencing was performed at the Washington University St. Louis Genome Technology Access Center generating in total 190 million unidirectional 50-bp reads using an Illumina HiSeq2000 instrument (Illumina).

#### RNA Seq of *E. faecalis* Isolated From SIHUMI Colonized Mice

*E. faecalis* cells in the colonic content of SIHUMI colonized wild type (*n* = 6 mice/group; colonization period: 16 weeks) and IL-10^−/−^ mice (*n* = 13 mice/group; colonization period: 16 weeks) were isolated by immuno-magnetic separation. Total bacterial RNA was isolated as described above and purified using the RNeasy MinElute Cleanup kit (Qiagen). Contaminating genomic DNA was eliminated using TURBO DNase (Thermo Fisher Scientific) and RNA integrity was validated by agarose gel electrophoresis. Ribosomal RNA was depleted by using the RiboMinus™ Transcriptome Isolation Kit (Bacteria, Thermo Fisher Scientific) according to the manufacturer's instructions. Subsequently, the RNA was fragmented for 180 s using a Covaris E220 sonicator set at 10% duty cycle, intensity of 5 and 200 cycles/burst in 120 μl 1 mM Tris-EDTA pH 8. The fragmented RNA was precipitated together with 1 μl glycogen (Thermo Fisher Scientific), 1:10 sample volume 3 M sodium acetate (Ambion) and 2.5 sample volume of 100% EtOH. Next, the RNA was dephosphorylated using 10 U Antarctic phosphatase per 300 ng RNA at 37°C for 30 min (New England Biolabs). The RNA fragments were recovered using the miRNeasy Mini kit (Qiagen) followed by 5'-phosphorylation using 20 U T4 polynucleotide kinase at 37°C for 60 min (Thermo Fisher Scientific). Again, the miRNeasy Mini kit was used for recovery. RNA fragment size was examined using the BioAnalyzer on an RNA Pico Chip (Agilent Technologies). The cDNA library was prepared as described in the TruSeq Small RNA Library Prep Reference Guide (Illumina) with the following exceptions: After adapter ligation and reverse transcription, fragments were size-selected between 145 and 300 bp corresponding to an insert length between 18 and 173 bp. The sequencing was conducted at the Core Facility Microbiome, generating in total 300 million unidirectional 50 bp reads using an Illumina HiSeq2000 instrument.

#### Bioinformatics

Data analysis was performed using the Galaxy web platform (50) (server: usegalaxy.eu). Quality of the raw sequence data was assessed with the FastQC tool (version 0.71). After adapter clipping with the Clip adapter sequences tool (version 1.0.3), Illumina FASTQ files were mapped to the reference genome of *E. faecalis* OG1RF (GenBank: NC_017316.1) using Bowtie2 (version 2.3.4.2). Using htseq-count, the aligned reads overlapping with genomic features were counted. Differentially expressed genes between bacteria isolated from inflamed IL-10^−/−^ vs. healthy wild type mice were determined using DEseq2 (version 2.11.40.2). KEGG gene set enrichment analysis was executed using the Bioconductor clusterProfiler package (version 3.8.1) implemented in R (51).

### Histopathological Analysis

The intestinal tract of gnotobiotic wild type and IL-10^−/−^ mice was removed immediately after sacrificing the animals and cleaned from stool. Cross-sections or “swiss-roles” (52) of parts from the intestinal tract were fixed in 4% formalin, dehydrated, and embedded in paraffin. For histopathological analysis, tissue samples were cut into 5 μm sections, deparaffinized and stained with hematoxylin and eosin. The histopathology was evaluated by an independent examiner in a blinded manner as described previously (53), resulting in scores from 0 (not inflamed) to 12 (highly inflamed).

### RNA Isolation (Mouse Tissue)

Tissue sections of distal colon were stored in RNAlater (Sigma Aldrich) at −80°C. Tissue samples were transferred into 500 μl RA1 buffer (Macherey Nagel) supplemented with 10 mM DTT (Sigma Aldrich) and the tissue was homogenized using a Miccra D-1 homogenizer. RNA was isolated using the NucleoSpin RNA Kit (Macherey Nagel) according to the manufacturer's instructions.

### Reverse Transcription and Gene Expression Analysis via qPCR (RNA From Mouse Tissue)

Complementary DNA (cDNA) was synthesized from 1 μg RNA using the Moloney murine leukemia virus (MMLV) Point Mutant Synthesis System (Promega) and random hexamers. Quantification was performed using the LightCycler 480 Universal Probe Library System (UPL, Roche). Primers (Sigma Aldrich) and corresponding UPL probes are listed in Table 4. Relative quantification of gene expression was calculated by means of 2^−ΔΔ*Ct*^ values: $2^{-\left[{\left(\text{C}{\text{t}}_{\text{target gene}}-\text{C}{\text{t}}_{\text{housekeeping gene}}\right)}^{\text{treated}}-{\left(\text{C}{\text{t}}_{\text{targetgene}}-\text{C}{\text{t}}_{\text{housekeepinggene}}\right)}^{\text{untreated}}\right]}$. Relative gene expression values were normalized to GAPDH and HPRT expression.

**Table 4:** Primers and corresponding UPL probes used for mouse gene expression analysis.

**Targeted gene**	**Gene ID (NCBI gene)**	**Oligonucleotide sequence (5'-3')**	**UPL probe**
GAPDH	14433	F: GGGTTCCTATAAATACGGACTGC R: CCATTTTGTCTACGGGACGA	52
HPRT	110286595	F: TCCTCCTCAGACCGCTTTT R: CCTGGTTCATCATCGCTAATC	95
TNFα	21926	F: TGCCTATGTCTCAGCCTCTTC R: GAGGCCATTTGGGAACTTCT	49
IFNγ	15978	F: GGAGGAACTGGCAAAAGGAT R: TTCAAGACTTCAAAGAGTCTGAGG	21
IL-12p40	16160	F: ATCGTTTTGCTGGTGTCTCC R: GGAGTCCAGTCCACCTCTACA	78
IL-17	16171	F: TGTGAAGGTCAACCTCAAAGTC R: AGGGATATCTATCAGGGTCTTCATT	50

### MLN Cell Cultures

MLNs were aseptically harvested from gnotobiotic wild type and IL-10^−/−^ mice associated with the SIHUMI consortium. Subsequently, tissue was homogenized and filtrated through 70 μm cell strainers (VWR) resulting in unfractionated single cell suspensions. MLN cells, isolated from individual mice (for IL-10^−/−^ mice) or a pool of mice (wild type mice), were cultured (5 × 10^5^ cells per well) in RPMI-1640 medium (Sigma Aldrich) containing 10% fetal calf serum (Biochrom) and 1% antibiotics/antimycotics (Sigma Aldrich) and stimulated with 15 μg/ml of bacterial lysate from the respective colonizing species. After incubation for 72 h, the supernatants were collected for cytokine analysis and stored at −80°C.

### Cytokine Quantification

IL-12p40 and IFNγ concentration in cell culture supernatant was quantified using commercially available ELISA kits (eBioscience).

### Ethanolamine Quantitation in Colonic Content by LC-MS/MS Analysis

Quantitation of ethanolamine in colonic content from wild type and IL-10^−/−^ mice monoassociated with *E. faecalis* OG1RF or Δ*eutVW* was performed at the Bavarian Center for Biomolecular Mass Spectrometry (BayBioMS, Freising, Germany). Sample preparation and mass spectrometry quantitation were modified after (54). Details are provided in the Supplementary Material.

### Statistical Analysis

Statistical analyses were performed using Prism version 6.05 (GraphPad). Unless otherwise stated, data are presented as mean ± standard deviation. Shapiro-Wilk or D'Agostino-Pearson omnibus test was used to assess the normality of distribution of investigated parameters. Statistical significant differences between two groups were determined using two-tailed unpaired parametric *t*-test. Statistical significant differences between three or more unpaired groups were determined using one-way ANOVA followed by pairwise testing (Tukey). Effects of genotype and colonization groups were compared using two-way ANOVA followed by pairwise testing (Tukey). Differences were considered significant for ^*^p ≤ 0.05, ^**^p ≤ 0.01, ^***^p ≤ 0.001, ^****^p ≤ 0.0001.

Analysis of RNA seq data was performed using Galaxy platform web platform (50) or R as described in detail above.

## Results

### *E. faecalis* Adapts to a Chronic Inflammatory Environment by Altering Its Gene Expression Profile

**Figure 1:**
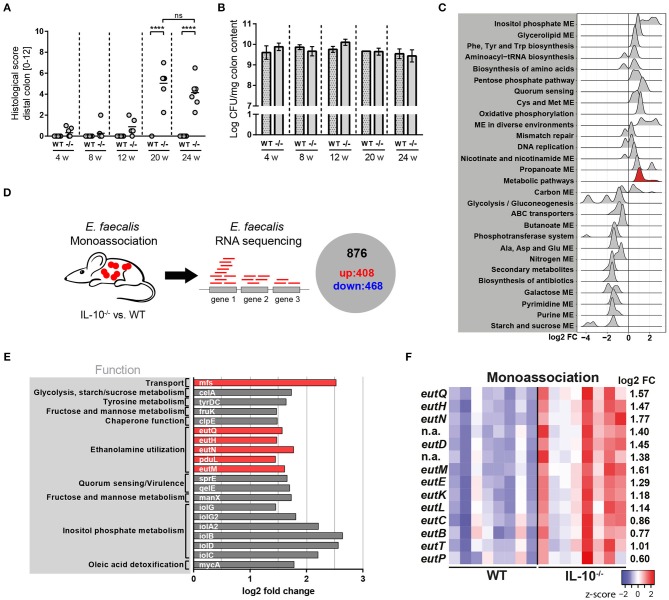
*E. faecalis* adapts to a chronic inflammatory environment by altering its gene expression profile. **(A,B)** Wild type (WT) and IL-10^−/−^ (–/–) mice were monoassociated with *E. faecalis* for 4, 8, 12, and 24 weeks (w). **(A)** Histological inflammation score in the distal colon. **(B)** *E. faecalis* counts/mg in luminal contents from colon. **(C)** Experimental setup: Transcriptional profiling of luminal bacteria isolated from the colon of *E. faecalis*-monoassociated IL-10^−/−^ (inflamed) and healthy wild type mice (*n* = 8/group) revealed 408 significantly up-regulated genes and 486 down-regulated genes in *E. faecalis* under inflammatory conditions. **(D–F)** Expression profiles of *E. faecalis* in an inflamed (IL-10^−/−^) vs. a non-inflamed (WT) environment in monoassociated mice. **(D)** Expression distribution (log2 lower or higher) of genes for GSEA-enriched categories (KEGG). The gene group containing *eut*-genes is highlighted red. **(E)** Top 20 up-regulated genes. Only annotated genes with known function are shown. Genes of the *eut* locus are highlighted in red. Gene functions were assigned according to KEGG database. **(F)** Differential expression of genes in relation to a chronically inflamed environment is shown for significantly regulated *eut* genes. The log2 ratio of mean abundance (IL-10^−/−^ vs. WT) of normalized expression levels is shown (up regulation is indicated red, down regulation is indicated blue). Differences were considered significant for ****p ≤ 0.0001.

To analyse the kinetics of *E. faecali*s-driven inflammation, we monoassociated germ-free wild type and IL-10^−/−^ mice at the age of 10 weeks with *E. faecalis* wild type OG1RF for different periods of time. After 4, 8, and 12 weeks of colonization, IL-10^−/−^ mice showed mild signs of inflammation in the distal colon as indicated by the histological score. Between the 12th and the 20th week of colonization, IL-10^−/−^ mice started to develop severe distal colonic inflammation reaching a plateau after week 20. A similar disease pattern was also observed in mid colon with an overall lower level of inflammation (Supplementary Figure 1A). In proximal colon, the inflammation persisted at a very low level throughout the analyzed colonization periods (Supplementary Figure 1B). All wild type mice remained disease-free (Figure 1A). The colonization density as detected by countable colony forming units (CFU) in luminal colon contents was not affected by the level of inflammation (Figure 1B). Based on the kinetic of distal colonic inflammation and on previous publications, 16 weeks of colonization were used in subsequent experiments. At this time, inflammation was still in the ascending phase and therefore advantageous for the analysis of factors modulating disease severity.

We hypothesized that *E. faecalis* quickly adapts to a chronic inflammatory environment by altering its gene expression profile. These adaptation mechanisms are likely responsible for the persistence and virulence of these bacteria in a susceptible host. To assess *E. faecalis* disease-associated transcriptome, we monoassociated mice with *E. faecalis* OG1RF for 16 weeks and performed RNA sequencing of bacteria isolated from luminal colon content of healthy wild type and inflamed IL-10^−/−^ mice. Transcriptional profiling identified 408 significantly up-regulated and 468 significantly down-regulated genes under conditions of chronic inflammation (Figure 1C and Supplementary Figure 1C, Supplementary Table 1). The samples clearly clustered according to mouse genotype as detected by principal component analysis (PCA) indicating that inflammation modulates *E. faecalis* gene expression (Supplementary Figure 1D). *E. faecalis* response to inflammation was characterized by an up regulation of genes associated with a bacterial stress response toward unfavorable growth conditions. KEGG pathway enrichment analysis showed an enrichment of the biosynthesis of amino acids, quorum sensing, mismatch repair, and oxidative phosphorylation among up-regulated genes (Figure 1D). Induction of these processes has also been observed in previous studies analyzing *E. faecalis* and *E. coli* adaptation during periods of stress (55–57). Nucleotide synthesis genes were in general down regulated, as indicated by an enrichment of purine- and pyrimidine metabolism transcripts in wild type mice. Furthermore, a large number of genes involved in metabolism and nutrient acquisition were differentially expressed. Sugar metabolism and transport were repressed with an enrichment of starch and sucrose metabolism, ABC transport, PTS transport and glycolysis among down-regulated genes. On the other hand, genes necessary for the utilization of alternative carbon sources were up regulated with an enrichment of inositol phosphate metabolism and glycerolipid metabolism (Figure 1D). Interestingly, genes of the ethanolamine utilization (*eut*) locus were also identified among the top 20 up-regulated genes in response to an inflammatory environment (Figure 1E). In total, 14 *E. faecalis eut* genes were significantly up regulated in inflamed IL-10^−/−^ mice indicating an important function of this metabolic pathway for the survival and virulence of *E. faecalis* in the inflamed intestine (Figure 1F).

### *E. faecalis* Ethanolamine Utilization Has Protective Functions in Gnotobiotic Il-10^−/−^ Mice Colonized With a Simplified Microbial Consortium

**Figure 2:**
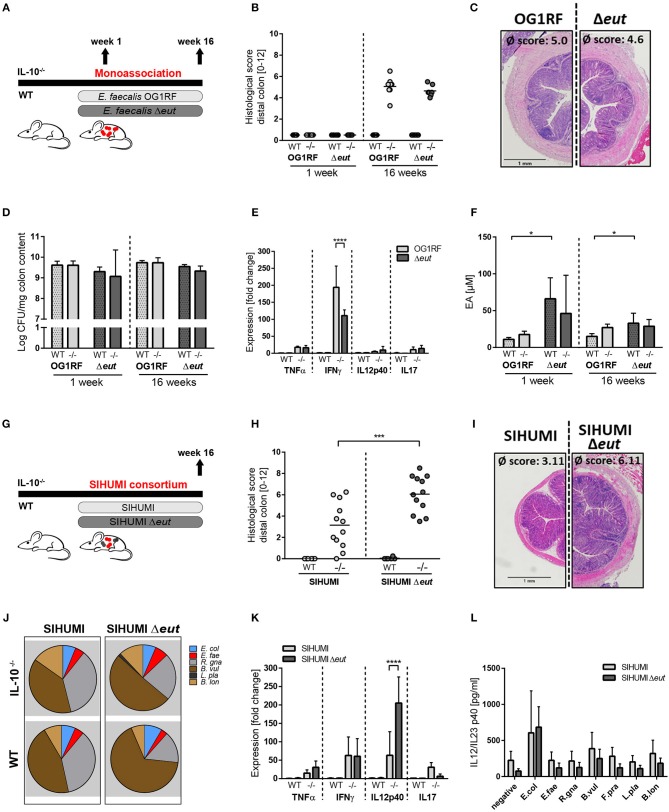
*E. faecalis* ethanolamine utilization has protective functions in gnotobiotic IL-10 ^−/−^ mice colonized with a simplified microbial consortium. **(A–F)** Germ-free wild type (WT) and IL-10^−/−^ (–/–) mice were monoassociated with *E. faecalis* wild type (OG1RF) or the Δ*eut* mutant for 1 and 16 weeks, respectively. **(A)** Experimental setup. **(B)** Histological inflammation in the distal colon. **(C)** Representative hematoxylin/eosin-stained sections of distal colon after 16 weeks of colonization. **(D)** *E. faecalis* presence in luminal contents from colon as log CFU counts/mg **(E)** Cytokine expression in colon tissue sections. Values are shown as fold-change normalized to cytokine expression levels in wild type mice colonized with *E. faecalis* wild type strain. **(F)** Quantitation of ethanolamine in luminal contents from colon. **(G–L)** Germ-free wild type (WT) and IL-10^−/−^ (–/–) mice were colonized with SIHUMI consortium including *E. faecalis* wild type (SIHUMI) or the Δ*eut* mutant (SIHUMI Δ*eut*) for 16 weeks. **(G)** Experimental setup. **(H)** Histological inflammation in the distal colon. **(I)** Representative hematoxylin/eosin-stained sections of distal colon after 16 weeks of colonization. **(J)** Relative abundances of SIHUMI bacterial species in luminal colon content by 16S rRNA gene targeted qPCR. **(K)** Cytokine expression in colon tissue sections shown as fold-change normalized to cytokine expression levels in wild type mice colonized with the SIHUMI consortium. **(L)** IL-12p40 secretion of MLN cells isolated from SIHUMI or SIHUMI Δ*eut* colonized mice that were re-activated with the respective bacterial lysate for 72 h. *E. col, Escherichia coli*; *E. fae, Enterococcus faecalis*; *R. gna, Ruminococcus gnavus*; *B. vul, Bacteroides vulgatus*; *L. pla, Lactobacillus plantarum*; *B. lon, Bifidobacterium longum*. Differences were considered significant for *p ≤ 0.05, ***p ≤ 0.001, ****p ≤ 0.0001.

To determine whether the utilization of ethanolamine (EA) is relevant for *E. faecalis* colonization and colitogenic activity, we monoassociated germ-free wild type and IL-10^−/−^ mice with *E. faecalis* wild type OG1RF or an Δ*eutVW* mutant strain (Δ*eut*) for 1 and 16 weeks (Figure 2A). The Δ*eut* strain does not express the *eut* genes and is not able to utilize EA (43, 58, 59). After 1 week of colonization, wild type and IL-10^−/−^ mice showed no signs of inflammation. After 16 weeks of colonization, IL-10^−/−^ mice developed severe inflammation in the distal colon, independent of the *E. faecalis* strain used for monoassociation (OG1RF histopathological score: 5.0 ± 0.9, Δ*eut* histopathological score: 4.6 ± 0.5). All wild type mice remained disease-free (Figures 2B,C). *E. faecalis* OG1RF and Δ*eut* mutant strain showed a similar colonization density as detected by CFU in luminal contents from colon (Figure 2D). The colon inflammation in IL-10^−/−^ mice was accompanied by increased spleen weights (Supplementary Figure 2A) and increased expression of pro-inflammatory markers in the distal colon tissue. The expression levels of TNF, IL-12p40, and IL-17 were similar for both colonization groups, only IFNγ expression levels were increased in *E. faecalis* wild type OG1RF compared to Δ*eut* mutant monoassociated IL-10^−/−^ mice (Figure 2E). To determine whether *E. faecalis* catabolizes EA *in vivo*, we measured EA concentrations in intestinal fluid. Luminal EA concentrations were significantly higher in wild type mice monoassociated with Δ*eut* mutant, indicating catabolism of EA by *E. faecalis* OG1RF. This trend was also observed in IL-10^−/−^ mice after 4 weeks of colonization, but after 16 weeks of colonization intestinal EA concentrations were similar in both *E. faecalis* OG1RF and Δ*eut*-mutant monoassociated mice (Figure 2F).

Despite considerable up regulation of *eut* genes, the deletion of ethanolamine utilization revealed no impact on *E. faecalis* colitogenic activity in monoassociated mice. To test the hypothesis that *E. faecalis* EA utilization is only important for bacterial fitness in competition with other microbes, we characterized the colitogenic activity of *E. faecalis* Δ*eut* mutant in the context of a simplified human microbial consortium (SIHUMI). We colonized germ-free wild type and IL-10^−/−^ mice with SIHUMI consortium either containing *E. faecalis* wild type OG1RF (SIHUMI) or *E. faecalis* Δ*eut* (SIHUMI Δ*eut*) mutant strain for a period of 16 weeks (Figure 2G). When we introduced the bacterial consortium to germ-free mice, 6 out of 7 species successfully colonized the mouse intestine, as determined by 16S rRNA gene-based quantitative qPCR (Figure 2J). *Faecalibacterium prausnitzii* could not be detected by qPCR and droplet digital PCR at any time (Figure 2J, Supplementary Figures 2B,C) in intestinal content (taken at week 4 and 16 of colonization) and feces (taken at week 2, 4, 8, and 16 of colonization). While IL-10^−/−^ mice colonized with the SIHUMI consortium gradually developed inflammation in the distal colon (SIHUMI histopathological score: 3.1 ± 2.3), colitis severity was significantly increased when *E. faecalis* OG1RF was replaced by Δ*eut* mutant strain (SIHUMI Δ*eut* histopathological score: 6.1 ± 1.8) (Figures 2H, I). These results surprisingly suggest protective functions of *E. faecalis* EA utilization in the context of chronic intestinal inflammation. Tissue pathology was accompanied by increased spleen weights in IL-10^−/−^ mice colonized with the SIHUMI Δ*eut* consortium (Supplementary Figure 2D). As expected, all colonized wild type mice remained disease-free. The replacement of *E. faecalis* wild type OG1RF strain by Δ*eut* mutant strain resulted in a shift of the bacterial community with a significant increase of *Bacteroides vulgatus* relative abundance accompanied by a decrease in *Ruminococcus gnavus* abundance (Figure 2J, Supplementary Figure 5). Interestingly, the *E. faecalis* Δ*eut* mutant strain showed an increased colonization density compared to the wild type OG1RF strain, as detected by CFU in luminal contents from colon (Supplementary Figure 2E). However, *E. faecalis* abundance did not correlate with the histopathology score of the distal colon (Supplementary Figure 2F). The only significant correlation between bacterial abundance and the level of colonic inflammation was detected for *R. gnavus* with a decreased abundance being associated with higher inflammatory scores (Supplementary Figures 2G, 5). Tissue sections from the distal colon of IL-10^−/−^ mice showed high expression of pro-inflammatory cytokines in comparison to wild type mice. Tissue expression of TNF and IL-12p40 reflected the degree of histopathology with higher expression levels in SIHUMI Δ*eut* colonized compared to SIHUMI colonized IL-10^−/−^ mice. Expression levels of IFNγ were similar between SIHUMI and SIHUMI Δ*eut* colonized IL-10^−/−^ mice, while IL-17 expression was decreased in SIHUMI Δ*eut* colonized IL-10^−/−^ mice (Figure 2K). To address the bacterial antigen-specific immune responses in colonized mice, we isolated MLN cells from IL-10^−/−^ mice colonized with SIHUMI or SIHUMI Δ*eut* consortium and re-stimulated them with respective lysates of each of the colonizing species. Unfractionated MLN cells produced high levels of IL-12p40 when stimulated with *E. coli* lysate, while IL-12p40 secretion in response to all other lysates was similar to the negative control. The level of MLN activation was similar for all *E. faecalis* strains (Figure 2L). Since replacement of *E. faecalis* wild type by the Δ*eut* mutant resulted in a more severe inflammatory phenotype, we investigated whether increased inflammation was associated with an enhanced humoral immune response against specific SIHUMI species. It has been demonstrated by others that certain commensal bacteria induce serum IgA responses that confer immunity against polymicrobial sepsis (60). When we probed lysates of SIHUMI species with sera from SIHUMI or SIHUMI Δ*eut* colonized IL-10^−/−^ mice, we observed the highest number of IgA immunoreactive bands for lysates of *B. vulgatus* and *R. gnavus*, the two most abundant species in colonized mice (Supplementary Figure 2H and Figure 2J). Similar results have been obtained for serum IgG antibodies (data not shown). The serum IgA response against SIHUMI species was not influenced by the level of inflammation, as the number and intensity of immunoreactive bands was similar for lysates probed with SIHUMI or SIHUMI Δ*eut* sera (Supplementary Figure 2H).

### Complex Bacterial Consortia Reprogram *E. faecalis* Gene Expression

**Figure 3:**
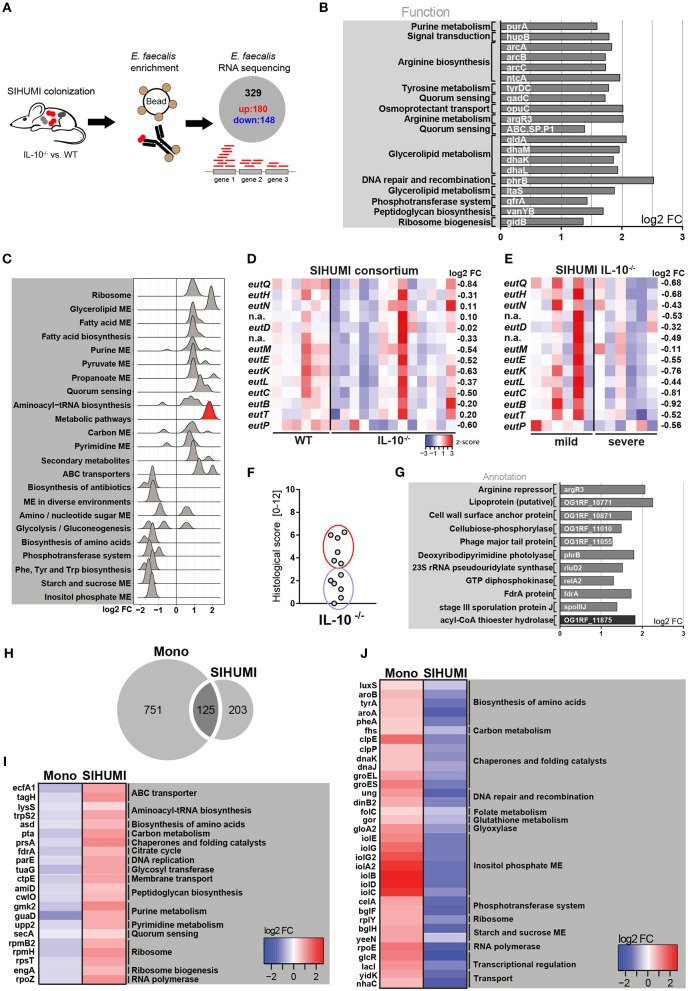
Complex bacterial consortia reprogram *E. faecalis* gene expression in response to inflammation. Luminal *E. faecalis* cells were isolated from the colon of inflamed IL-10^−/−^ (*n* = 13) and healthy wild type (WT) (*n* = 6) mice, colonized with SIHUMI consortium (16 weeks), using immuno-magnetic separation and subjected to RNA sequencing analysis. **(A)** Experimental setup: Transcriptional profiling revealed 180 significantly up-regulated and 148 significantly down-regulated genes in *E. faecalis* isolated from IL-10^−/−^ mice compared to *E. faecalis* isolated from wild type mice colonized with the SIHUMI consortium. **(B)** Top 20 up-regulated genes in *E. faecalis* isolated from SIHUMI colonized mice (IL-10^−/−^ vs. WT). Only annotated genes with known function are shown. Gene functions were assigned according to KEGG database. **(C)** Expression distribution of enriched genes for GSEA enriched categories (KEGG) in *E. faecalis* isolated from SIHUMI colonized mice (IL-10^−/−^ vs. WT). The gene group highlighted red contains the *eut*-genes. **(D)** Differential expression of *eut* genes in *E. faecalis* of an inflamed vs. non-inflamed environment. The log2 ratios of mean abundance (IL-10^−/−^ vs. WT) of normalized expression levels are shown (up regulation is indicated red, down regulation is indicated blue). **(E)** Differential expression of *eut* genes in *E. faecalis* of a severely inflamed vs. mildly inflamed environment. The log2 ratios of mean abundances of normalized expression levels are shown (up regulation is indicated red, down regulation is indicated blue). **(F)** Histological inflammation scores in the distal colon of SIHUMI colonized IL-10^−/−^ mice comparing severely inflamed mice (red) and mildly inflamed mice (blue). **(G)** Significantly differentially regulated genes of *E. faecalis* isolated either from severely inflamed or mildly inflamed SIHUMI colonized IL-10^−/−^ mice (up regulation is indicated gray, down regulation is indicated black). **(H–J)** Differentially regulated genes in response to inflammation of *E. faecalis* isolated from monoassociated (IL-10^−/−^ vs. WT) compared to SIHUMI-colonized mice (IL-10^−/−^ vs. WT). **(H)** Venn diagram showing the differentially regulated genes shared. **(I)** Genes down regulated in monoassociated and up regulated in SIHUMI-associated mice, respectively. The log2 ratios of mean abundances of normalized expression levels are shown (up regulation is indicated red, down regulation is indicated blue). Gene functions were assigned according to the KEGG database; only annotated genes with known function are shown. **(J)** As before, but genes up regulated in monoassociated and down regulated in SIHUMI associated mice, respectively.

Since we observed a protective effect of *E. faecalis* EA utilization only in a complex bacterial community and not under conditions of *E. faecalis* monoassociation, we aimed to gain a better understanding of *E. faecalis* gene expression in the presence of other microbes. We purified *E. faecalis* cells from the colon content of SIHUMI colonized wild type and IL-10^−/−^ mice using immuno-magnetic separation and performed RNA sequencing (Figure 3A). Transcriptional profiling identified 180 significantly up- and 148 significantly down-regulated genes under inflammatory conditions (Supplementary Figure 3A). The samples clustered according to mouse genotype, as shown by PCA analysis (Supplementary Figure 3B). Among the top 20 up-regulated genes, several genes associated with arginine biosynthesis and glycerol metabolism were identified (Figure 3B). Arginine biosynthesis genes have been shown to be up-regulated in lactic acid bacteria in response to acid stress (55) and genes involved in glycerol metabolism have been shown to be induced during *E. faecalis*-mediated mouse peritonitis as well as intestinal colonization (61, 62). Additionally, an *E. faecalis* mutant affected in glycerol metabolism showed attenuated virulence in a mouse peritonitis model (62).

Surprisingly, we observed no overlap with the top up-regulated genes in *E. faecalis* isolated from monoassociated mice (Figures 3B, 1E). Additionally, the functional categories enriched among all differentially regulated genes were mainly different between *E. faecalis* monoassociation or in the context of the SIHUMI community (Figures 3C, 1D). In the SIHUMI consortium, *E. faecalis* response to inflammation was characterized by an induction of cellular pathways required for growth and replication, including ribosome, aminoacyl-tRNA biosynthesis, nucleotide synthesis, pyruvate and propanoate metabolism, ABC transport and fatty acid biosynthesis and metabolism. In contrast to *E. faecalis* monoassociation, inositol phosphate metabolism and the biosynthesis of amino acids were repressed. Quorum sensing and glycerolipid metabolism were induced, whereas phosphotransferase systems and starch and sucrose metabolism were repressed in both mono- and complex association of IL-10^−/−^ mice (Figures 3C, 1D). Interestingly, *E. faecalis* isolated from SIHUMI colonized mice showed higher expression of *eut* genes in healthy (Figure 3D) and mildly inflamed (Figure 3E) compared to inflamed animals.

In IL-10^−/−^ mice colonized with the SIHUMI consortium, we observed a gradient of colitis severity. Six out of 12 mice had a colitis score <3 and were defined as mildly inflamed animals, the remaining 6 mice with a colitis score >3 were defined as severely inflamed animals (Figure 3F). When comparing *E. faecalis* gene expression between severely and mildly inflamed IL-10^−/−^ mice, only 11 genes showed statistical significant regulation (Supplementary Figures 3C,D). In particular, the lipoprotein SpoIIIJ (OG1RF_12576), which has been shown to be important for bile resistance of *E. faecalis* (63) and a cell wall surface anchor family protein (OG1RF_10871), which is associated with *E. faecalis* biofilm formation (64), appeared to be interesting candidates for *E. faecalis* virulence (Figure 3G).

When comparing *E. faecalis* transcriptional response to inflammation between bacteria isolated from mono- and complex-associated mice on the gene level, the majority of differentially regulated genes were not shared. This suggests that the bacterial environment reprograms *E. faecalis* gene expression. Only 125 genes were shared between both colonization environments, whereas 751 genes were exclusively regulated in monoassociation and 203 genes were exclusively regulated in the SIHUMI community (Figure 3H). In addition, the majority of shared genes showed opposite patterns of regulation. Several genes associated with fundamental cellular pathways were down regulated in *E. faecalis* monoassociated mice, whereas these genes were strongly up regulated in *E. faecalis* of the SIHUMI community (Figure 3I). This includes genes associated with the translation apparatus (*lysS, trpS2, rpmB2, rpmH, rpsT, engA*), RNA synthesis (*rpoZ*), DNA replication (*parE*), citrate cycle (*fdrA*), peptidoglycan biosynthesis (*amiD, cwlO*), nucleotide metabolism (*gmk2, guaD, upp2*), and transport (*ecfA1, tagH, ctpE, secA*) (Figure 3I). *Vice versa*, we observed an up regulation of several stress-response genes in *E. faecalis* of monoassociated mice, including genes that encode for proteases and chaperones (*clpE, clpP, dnaK, dnaJ, groEL, groES*), a ribosomal stress protein (*rplY*), a detoxifying enzyme (*gloA2*) and proteins associated with DNA repair (*ung, dinB2*) and oxidative stress resistance (*gor*) (55, 65). In addition, genes associated with the biosynthesis of amino acids (*luxS, aroA, aroB, tyrA, pheA*), transport (*yidK, nhaC*), transcriptional regulation (*glcR, lacI*) and a protein homologous to RNA polymerase subunit RpoE, were up-regulated in *E. faecalis* of monoassociated mice, but down-regulated in *E. faecalis* SIHUMI colonized mice (Figure 3J).

### Complex Microbial Consortia Interactions Reprogram *E. faecalis* Colitogenic Activity

**Figure 4:**
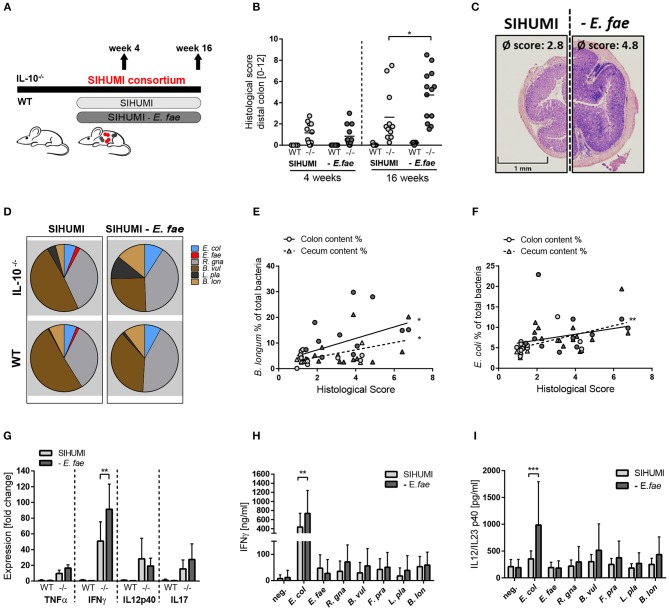
Complex microbial consortia interactions reprogram colitogenic activity of *E. faecalis* in gnotobiotic IL-10^−/−^ mice. **(A)** Experimental setup: Germ-free IL-10^−/−^ (–/–) and wild type (WT) mice were colonized with SIHUMI consortium or SIHUMI consortium without *E. faecalis* (*- E. fae*) for a period of 4 and 16 weeks. **(B)** Histological inflammation in the distal colon. **(C)** Representative hematoxylin/eosin-stained sections of distal colon after 16 weeks of colonization. **(D)** Relative abundances of SIHUMI bacterial species in luminal colon content by 16S rRNA gene targeted qPCR. **(E)** Correlation between *B. longum* abundance (colon and cecum luminal content) and mean histological scores of cecum tip and distal colon. **(F)** As before, correlation of *E. coli* abundance. **(G)** Cytokine expression in colon tissue sections shown as fold change, normalized to cytokine expression levels in wild type mice colonized with the SIHUMI consortium. **(H)** IFNγ secretion of MLN cells isolated from SIHUMI or SIHUMI without *E. faecalis* colonized mice (colonization period: 16 weeks) that were re-activated with the respective bacterial lysate for 72 h. **(I)** As before, IL-12p40 secretion. *E. col, Escherichia coli*; *E. fae, Enterococcus faecalis*; *R. gna, Ruminococcus gnavus*; *B. vul, Bacteroides vulgatus*; *L. pla, Lactobacillus plantarum*; *B. lon, Bifidobacterium longum*. Differences were considered significant for *p ≤ 0.05, **p ≤ 0.01, ***p ≤ 0.001.

We next addressed the question of whether the transcriptional differences of *E. faecalis* in mono- or complex-associated mice have functional consequences by applying the SIHUMI consortium with or without *E. faecalis* (SIHUMI-*E. fae*). Wild type and IL-10^−/−^ mice were colonized at the age of 10 weeks for a period of 4 or 16 weeks (Figure 4A). Surprisingly, the colonization of IL-10^−/−^ mice with SIHUMI in the absence of *E. faecalis* induced an aggravated inflammatory host response compared to the control. After 16 weeks of colonization, IL-10^−/−^ mice colonized with SIHUMI-*E. fae* consortium developed severe inflammation in the distal colon (score: 4.8 ± 2.4), while the degree of colitis was significantly reduced in IL-10^−/−^ mice colonized with SIHUMI in the presence of *E. faecalis* (score: 2.8 ± 2.5) (Figures 4B,C). After 4 weeks of colonization, IL-10^−/−^ mice showed mild pancolitis independent of the colonizing bacteria. All colonized wild type mice remained disease-free (Figures 4B,C). The histological inflammation was accompanied by increased spleen weights (Supplementary Figure 4A).

The absence of *E. faecalis* resulted in changes of the relative abundances of specific SIHUMI species in the colonic content. Compared to SIHUMI colonized mice, the relative concentrations of *E. coli* and *B. longum* were slightly higher, while *B. vulgatus* concentrations were lower in SIHUMI-*E. fae* colonized mice (Figure 4D, Supplementary Figure 6). In addition, the relative abundance of *E. coli* and *B. longum* correlated positively with the development of histopathology. *E. faecalis* abundance was not correlated with colitis activity (Figures 4E,F, Supplementary Figure 4D). The expression of the pro-inflammatory cytokine IFNγ was significantly increased in IL-10^−/−^ mice colonized with SIHUMI-*E. fae*, supporting the observation at the level of tissue pathology. TNF and IL-17 expression levels followed the same pattern, whereas IL-12p40 expression was not influenced by the composition of the colonizing consortium (Figure 4G). To address the bacterial antigen-specific immune responses of colonized mice, we isolated MLN cells from IL-10^−/−^ mice colonized with SIHUMI in the presence and absence of *E. faecalis* and re-stimulated them with respective lysates of each of the colonizing SIHUMI species. After 4 weeks of colonization, the secretion of IFNγ and IL-12p40 in response to *E. coli* lysate stimulation was slightly increased in MLN cells isolated from mice colonized with SIHUMI. All other lysates failed to activate the MLN cell cultures (Supplementary Figures 4B,C). After 16 weeks of colonization, MLN cells produced maximal levels of IFNγ and IL-12p40 upon stimulation with *E. coli* lysate, although the relative luminal concentrations of this bacterium were quite low (Figures 4D,H,I). The MLN response toward *E. coli* lysate was even more pronounced in MLNs isolated from mice colonized with SIHUMI—*E. fae* consortium (Figures 4H,I), suggesting compensatory mechanisms in the absence of *E. faecalis*. The level of serum IgA antibodies against SIHUMI species was only slightly influenced by the composition of the bacterial consortium. When we probed lysates of SIHUMI species with sera from SIHUMI or SIHUMI-*E. fae* colonized IL-10^−/−^ mice, we observed the highest number of immunoreactive bands for lysates of *B. vulgatus, R. gnavus*, and *E. coli*. The number and intensity of immunoreactive bands against *E. coli* and *B. vulgatus* lysates were slightly increased when probed with SIHUMI-*E. fae* sera, indicating increased levels of IgA antibodies against these species in mice colonized with SIHUMI in the absence of *E. faecalis* (Supplementary Figure 4E).

## Discussion

In this study, we demonstrate that *E. faecalis* functionally adapts to chronic inflammation, supporting the hypothesis that host-related mechanisms contribute to the colitogenic activity of opportunistic pathogens. *E. faecalis* is a disease-relevant pathobiont and colonization of IBD-related mouse models, such as IL-10^−/−^ mice, supports the concept that commensal bacteria harboring pathogenic traits drive chronic inflammation in genetically susceptible but not wild type hosts (66). In addition to previous identified genes modulating the colitogenic effect of *E. faecalis* (28), we performed transcriptional profiling of *E. faecalis* under inflammatory conditions and identified 876 genes to be differentially regulated compared to normal conditions. Despite considerable up regulation of 14 genes of the *eut* locus in inflamed IL-10^−/−^ mice, the deletion of *E. faecalis* ethanolamine utilization (Δ*eutVW*) showed no effect on the colonization density and colitogenic activity of *E. faecalis* in monoassociated mice. Interestingly, *E. coli* responds to inflammation in monoassociated IL-10^−/−^ mice by the up regulation of stress response genes, but targeted deletion of these genes aggravated the inflammatory phenotype (11, 12). This supports our observation that bacterial adaptation processes correlating with intestinal inflammation are not necessarily harmful for the host. In line with this, we have shown in a previous study that genes that produce two colitis-relevant structures in *E. faecalis* are not transcriptionally regulated in inflamed monoassociated IL-10^−/−^ mice (28). The measurement of luminal intestinal EA allowed us to demonstrate that *E. faecalis* utilizes this substrate *in-vivo* in wild type mice and not-inflamed IL-10^−/−^ mice. However, EA concentrations in inflamed IL-10^−/−^ mice were similar for both *E. faecalis* wild type OG1RF and Δ*eut* mutant colonization. This is surprising as *E. faecalis eut* genes were massively up regulated in inflamed IL-10^−/−^ mice relative to wild type controls. This paradox could be explained by limitations of our model as we only analyzed gene expression and not *eut* protein levels. Additional studies to understand *E. faecalis*' *eut*-gene expression and *in-vivo* EA metabolism are needed.

We provide for the first time evidence for a protective effect of bacterial EA utilization in the context of chronic intestinal inflammation. Using a simplified microbial consortium based on human strains (SIHUMI), we demonstrate that *E. faecalis* EA utilization attenuates colitis in mice colonized with a complex bacterial community. The replacement of *E. faecalis* wild type strain by a Δ*eut* mutant resulted in exacerbated colitis in SIHUMI colonized IL-10^−/−^ mice, as indicated by increased histological inflammation, increased tissue expression of TNF and IL-12p40 and increased spleen weights. The colonization density of Δ*eut* mutant was slightly increased compared to wild type *E. faecalis*. However, the relative abundance of *E. faecalis* did not correlate with the histological inflammation, suggesting that increased *E. faecalis* Δ*eut* numbers do not explain the aggravated inflammatory phenotype. MLN cells secreted high levels of IL-12p40 in response to *E. coli* lysate, but the highest IgA immunoreactivity was observed for *B. vulgatus* and *R. gnavus*, the two most abundant species. The bacterial community changed when *E. faecalis* wild type OG1RF was replaced by Δ*eut* mutant strain, with a significant increase in the relative abundance of *B. vulgatus* accompanied by a decrease in the abundance of *R. gnavus*. *B. vulgatus* induces colitis in HLA/B27-β_2_m rats, but does not induce inflammation in other colitis models (e.g., IL-10^−/−^ or IL-2^−/−^ mice) (67), which makes it difficult to assess whether this bacterium has colitogenic activity in a complex community in IL-10^−/−^ mice. Taken together, our data suggest that *E. faecalis* is not the driver of inflammation in the SIHUMI community, but modulates disease by interaction with co-colonizing bacteria. The catabolism of EA by *E. faecalis* may prevent the utilization of this metabolite by the more colitogenic species *E. coli*. It has been shown that EA can promote *E. coli* growth and virulence (35, 54). However, the expression of selected *E. coli* virulence (*fliC, fimH, ompA, ompC*) and ethanolamine utilization genes (*eutR, eutC, eutQ*) was not affected in our model (data not shown) and also the relative abundance of *E. coli* was not increased in the presence of *E. faecalis* Δ*eut* mutant.

Our data provide new insights into the role of EA utilization in microbiota-host interactions. EA plays a well-recognized role in host adaption and virulence of enteric pathogens including EHEC, *Salmonella* and *Listeria monocytogenes* (35, 36, 54, 68, 69). In contrast, we show that *E. faecalis* EA utilization has a protective function in IL-10^−/−^ mice in the presence of other resident bacterial species. These discrepancies might arise from differences in bacterial physiology and pathogenicity. The pathogen *Salmonella* requires alternative electron acceptors released from inflamed host tissue for EA utilization (34, 70), whereas *E. faecalis* can grow anaerobically on EA in the absence of alternative electron acceptors (71). In addition, others have demonstrated a protective effect of EA utilization by opportunistic pathogens as well. The metabolism of EA resulted in reduced virulence together with a delayed onset of disease in a hamster model of *Clostridium difficile* infection (72). Furthermore, EA utilization by commensal *E. coli* isolates allowed them to outcompete the pathogen EHEC (41). Consistent with our data, the colonization efficiency of *E. faecalis* Δ*eutVW* in the murine gastrointestinal tract was increased relative to the wild type strain (73). This stands in contrast to enteric pathogens, where the loss of ethanolamine utilization resulted in a competitive disadvantage (34, 36, 54). In *Caenorhabditis elegans*, an *E. faecalis eut* mutant was attenuated in virulence, but in this host some *E. faecalis* strains are regarded as a pathogenic organism resulting in the death of infected nematodes (38). In contrast, probiotic *E. faecalis* protect *C. elegans* against EHEC, underlining the importance of strain-level based experiments (74). In any case, the interesting correlation between bacterial pathogenicity and the role of EA utilization for bacterial survival and virulence awaits further investigation.

To the best of our knowledge, this is the first published study to analyze the influence of co-colonization with a bacterial consortium on gene expression and function of an opportunistic pathogen. Most importantly, we demonstrate that complex bacterial consortia interactions reprogram the gene expression profile and the colitogenic activity of *E. faecalis* toward a protective function. Transcriptional interactions between bacteria have already been illustrated for two members of the dominant gut bacterial phyla. *Eubacterium rectale* and *Bacteroides thetaiotaomicron* adapt their profile of utilized substrates in response to the co-colonizing species in gnotobiotic mice (75). In addition, Plichta et al. (76) demonstrated transcriptional species-specific interactions in a complex bacterial community with central metabolisms being strongly affected by coexistence with other microbes. Using immuno-magnetic separation, we were able to isolate *E. faecalis* cells from SIHUMI colonized mice. This allowed us to study *E. faecalis* transcriptional response to host inflammation depending on the microbial environment. Interestingly, the majority of regulated genes were not shared between *E. faecalis* colonizing in monoassociation or in the SIHUMI community. This shows that *E. faecalis* adapts transcriptionally to the co-colonizing microbes.

In monoassociated IL-10^−/−^ mice, *E. faecalis* gene expression was characterized by an enrichment of functions important for bacterial stress adaptation. General stress-response genes, amino acid biosynthesis genes and genes involved in the utilization of alternative carbon sources were up regulated. A similar regulation pattern has been observed by others analyzing *E. faecalis* transcriptome during mammalian infection. The intraperitoneal transcriptome of *E. faecalis* in gnotobiotic mice is characterized by the up regulation of stress-response genes and a metabolic adaptation to an inflamed environment (62). Frank et al. (77) demonstrated that *E. faecalis* cells undergo transcriptional adaptation and exist in a stringent response state in a rabbit subdermal abscess model. The stringent response, a bacterial stress adaptation mechanism, is characterized by the repression of fundamental cellular pathways, whereas amino acid biosynthesis genes and stress-survival genes are strongly induced. In addition, metabolic rearrangements including the utilization of alternative carbon sources are a hallmark of the stringent response (65, 78). Since we observe similar pathways regulated in response to inflammation and a link between *E. faecalis* stringent response and virulence has been demonstrated in a study by Gaca et al. (65), one could speculate that the gene expression of *E. faecalis* explains the colitogenic character of this bacterium in monoassociated IL-10^−/−^ mice. The detailed investigation of *E. faecalis* genes and their biological role in the pathophysiology of chronic inflammation awaits further investigation.

In co-colonization with other microbes, however, *E. faecalis* gene expression in inflamed mice is not primarily characterized by the induction of stress-response pathways, but rather by an enrichment of pathways important for bacterial growth and replication. Although some genes important for bacterial adaptation to environmental stress were up regulated (e.g., arginine deaminase pathway genes, *opuC, phrB*) (38, 55), the transcriptional profile does not indicate a stringent response state as observed in monoassociated *E. faecalis*. Fundamental cellular pathways like replication and translation were induced and the expression of general stress-response genes like *clpE, clpP, dnaK, dnaJ, groEL*, and *groES* were repressed (62, 79).

Previous studies analyzing *E. faecalis*' function in chronic inflammation were conducted in monoassociated IL-10^−/−^ mice and revealed a colitogenic behavior (23, 24, 27, 28). By characterizing *E. faecalis* as part of a microbial consortium, we are adding new knowledge to the complex interdependence of opportunistic pathogens, the genetically predisposed host and the luminal microbial environment. Colonization of IL-10^−/−^ mice with SIHUMI under omission of *E. faecalis* or in the presence of *E. faecalis* with defective EA utilization resulted in a more severe phenotype as indicated by the histological inflammation, spleen weights and the pro-inflammatory cytokine expression in colon tissue. This supports the hypothesis that *E. faecalis* provides protective mechanisms in complex consortia by the utilization of EA. A massive pro-inflammatory response of reactivated MLN cells to *E. coli* lysate stimulation and a positive correlation between histological score and *E. coli* abundance point to this bacterium and not *E. faecalis* as a main driver of SIHUMI mediated colitis. One could speculate that *E. faecalis* outcompetes *E. coli* or other SIHUMI strains for EA, but when *E. faecalis* is missing, or *E. faecalis* EA utilization is defective, the virulence of *E. coli* or other EA-responsive strains is enhanced leading to an increased immune response and inflammation.

Our data emphasize the dualistic character of *E. faecalis* and show that the colitogenic function of a bacterial strain is not only defined its gene repertoire, but also by co-colonizing microbes. Simultaneous colonization of IL-10^−/−^ mice with *E. coli* and *E. faecalis* resulted in more aggressive colitis as compared to disease induced by each species individually (80). Furthermore, *Helicobacter hepaticus* and *Lactobacillus reuteri* do not induce disease in monoassociated IL-10^−/−^ mice, but when both strains are used in a dual-association setup they induce severe colitis (81). The infection of Rag2-deficient and wild type mice with *Klebsiella pneumoniae, Proteus mirabilis*, or a combination of both strains triggered inflammation in the presence of a complex specific-pathogen free (SPF) microbiota, but *K. pneumoniae* and *P. mirabilis* dual-association of germ-free mice did not result in the development of colitis (82). Similarly, in the severe combined immunodeficiency mouse model, segmented filamentous bacteria were only effective in triggering intestinal inflammation in combination with a complex SPF microbiota (83). *Vice versa, E. coli* induces colitis in gnotobiotic IL-2-deficient mice, but co-association with *B. vulgatus* prevents colitis development (84).

In summary, we demonstrate that *E. faecalis* gene expression and colitogenic activity is influenced by co-colonizing microbes. A protective effect of *E. faecalis* EA utilization was only apparent in SIHUMI colonized mice, but not under monocolonized conditions. Additionally, we report a shift of phenotype from a colitogenic to a protective activity of *E. faecalis* in the IL-10^−/−^ mouse model depending on the bacterial environment. This model study with manageable bacterial complexity shows that monoassociation studies do not necessarily allow conclusions to be drawn about bacterial colitogenicity in IBD. This underlines that IBD is a complex disease in which both different host genes and bacterial genes play a role. The gradual development of a human-oriented complexity in carefully selected microbial consortia could be a viable way to unravel the complex microbe-host and microbe-microbe interactions in disease etiology. This may contribute to a basic understanding of the pathogenesis of human IBD and have therapeutic options.

## Data Availability

The RNA seq datasets generated for this study can be found in the SRA archive of NCBI under the accession No. PRJNA527872 and PRJNA528098.

## Ethics Statement

All animal procedures were approved by the Institutional Animal Care and Use Committee of the University of North Carolina, Chapel Hill, NC, USA or the Committee on Animal Health and Care of the local government (Regierung von Oberbayern, AZ 55.2-1-54-2532-109-2015) in Germany.

## Author Contributions

IL, IS, RS, and DH conceived and designed the experiments. IL, IS, and JH performed mouse experiments and tissue analyses. KN and JJH supported RNA sequencing work. KK and TH supported LC-MS/MS analysis. JJH, KK, KN, TH, RS, and DH contributed reagents, materials, or analysis tools. IL and DH wrote the manuscript, with edits by RS, IS, JJH, and KN.

### Conflict of Interest Statement

The authors declare that the research was conducted in the absence of any commercial or financial relationships that could be construed as a potential conflict of interest.
