# Supplementary material for: Complex Bacterial Consortia Reprogram the Colitogenic Activity of Enterococcus faecalis in a Gnotobiotic Mouse Model of Chronic, Immune-Mediated Colitis
Source: Front Immunol. 2019 Jun 20;10:1420. doi: 10.3389/fimmu.2019.01420 (PMC6596359; doi:10.3389/fimmu.2019.01420)
Supplement: Supplementary file 3 [file Data_Sheet_1.PDF]

Supplementary Material

1 Supplementary Figures

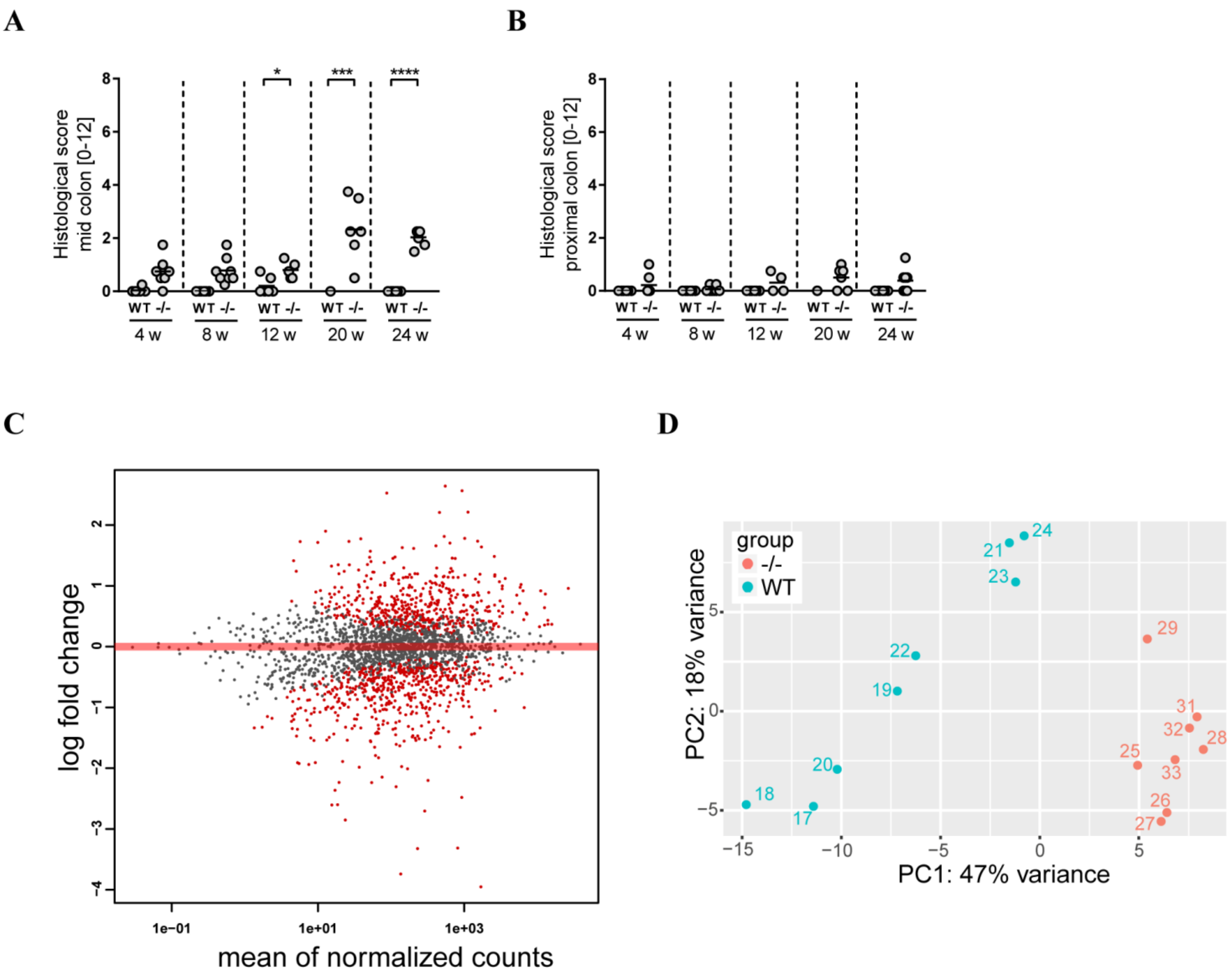

Supplementary Figure 1. (A) Histological inflammation in the mid and **(B)** proximal colon of wild type (WT) and IL-10$^{-/-}$ mice monoassociated with *E. faecalis* (colonization time: 4, 8, 12, 20 and 24 weeks). **(C)** Differentially expressed genes in *E. faecalis* isolated from monoassociated IL-10$^{-/-}$ vs. WT mice. The MA plot shows the distribution of log2 fold-changes in gene expression, the average of the counts normalized by size factor is shown on the x-axis. Each gene is represented with a dot. Genes with an adjusted p-value equal to or below 0.05 are shown in red. **(D)** PCA plot showing sample-to-sample distances according to their gene expression profiles for *E. faecalis* isolated from monoassociated IL-10$^{-/-}$ vs. WT mice. Differences were considered significant for *$p \leq 0.05$, ***$p \leq 0.001$, ****$p \leq 0.0001$.

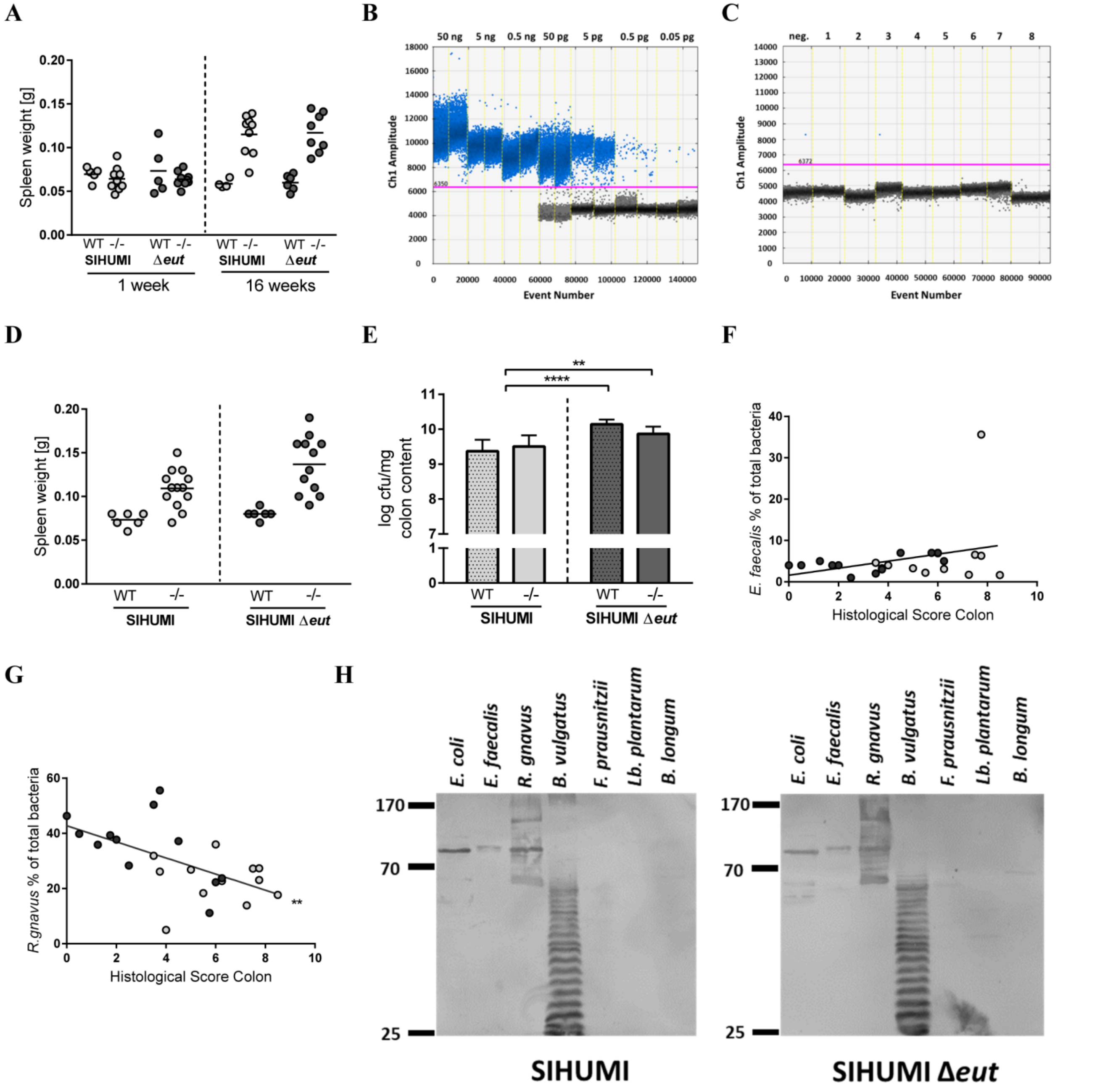

Supplementary Figure 2. (A) Spleen weight of wild type (WT) and IL-10$^{-/-}$ mice monoassociated with *E. faecalis* WT or the Δ*eut* mutant for 1 and 16 weeks. **(B, C)** Droplet digital PCR: PCR positive droplets are color-coded in blue, primers are targeted against the *F. prausnitzii* 16S rRNA gene. **(B)** Standard curve with *F. prausnitzii* DNA isolated from culture, DNA concentration per reaction is shown on top. **(C)** Droplet digital PCR reaction with 50 ng of total DNA isolated from luminal colon content of germfree (neg.) and SIHUMI colonized IL-10$^{-/-}$ (lanes 1-4) and WT (lanes 5-8) mice after a colonization period of 4 (lanes 1, 2, 5, and 6) and 16 weeks (lanes 3, 4, 7, and 8). **(D-H)** Germfree WT and IL-10$^{-/-}$ mice were colonized with SIHUMI consortium including *E. faecalis* OG1RF (SIHUMI) or the Δ*eut* mutant (SIHUMI Δ*eut*) for 16 weeks. **(D)** Spleen weight. **(E)** *E. faecalis* presence in luminal contents from colon shown as CFU counts/ml. **(F)** Correlation between *E.*

faecalis and **(G)** *R. gnavus* abundance (luminal colon content) and histological scores of distal colon. **(H)** Serum IgA against SIHUMI species: Western blots of the indicated bacterial lysates using sera from SIHUMI or SIHUMI Δ*eut* colonized IL-10$^{-/-}$ mice. Differences were considered significant for $^{**}p \leq 0.01$, $^{****}p \leq 0.0001$.

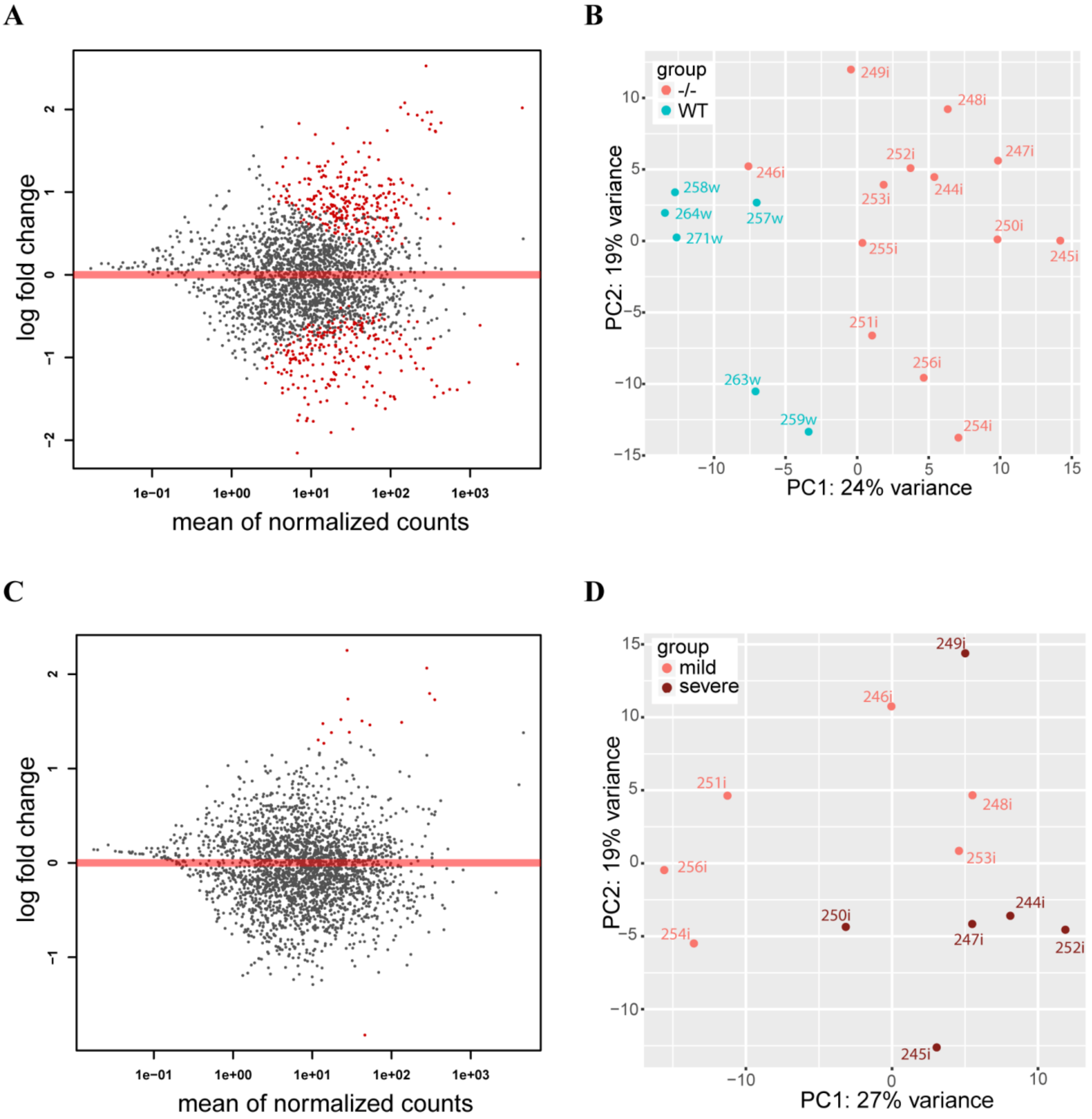

Supplementary Figure 3. (A, B) Differentially expressed genes in *E. faecalis* isolated from SIHUMI colonized IL-10$^{-/-}$ vs. wild type (WT) mice. **(A)** MA plot showing the distribution of log2 fold-changes in the gene expression, the average of the counts normalized by size factor is shown on the x-axis. Each gene is represented with a dot. Genes with an adjusted p-value equal to or below 0.5 are shown in red. **(B)** PCA plot showing sample-to-sample distances according to their gene expression profiles. **(C, D)** Differentially expressed genes in *E. faecalis* isolated from SIHUMI colonized

severely inflamed IL-10$^{-/-}$ vs. mildly inflamed IL-10$^{-/-}$ mice. **(C)** MA plot showing the distribution of log2 fold changes in the gene expression; the average of the counts normalized by size factor is shown on the x-axis. Each gene is represented with a dot. Genes with an adjusted p-value equal to or below 0.5 are shown in red **(D)** PCA plot showing sample-to-sample distances according to their gene expression profiles.

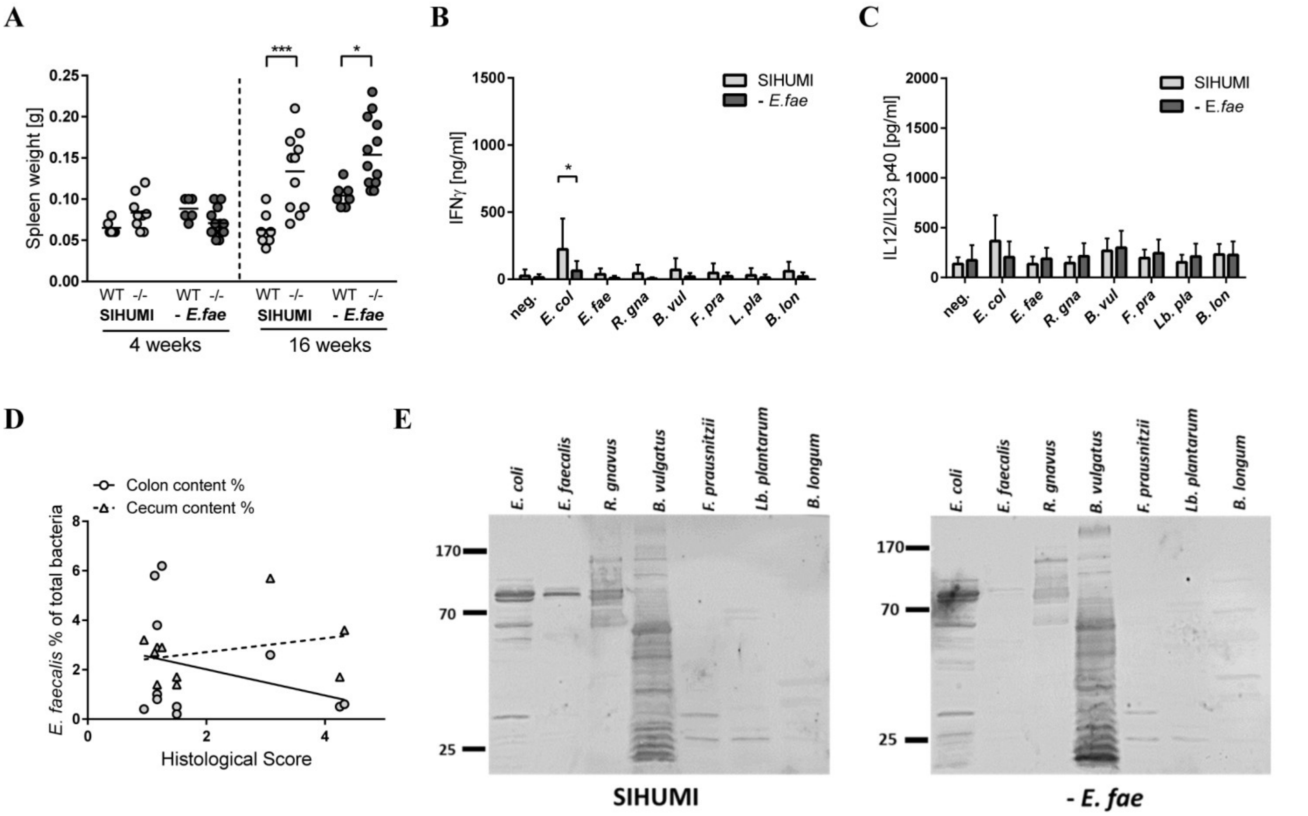

Supplementary Figure 4. Germfree IL-10$^{-/-}$ and wild type (WT) mice were colonized with SIHUMI consortium or SIHUMI consortium without *E. faecalis* (- *E. fae*) for a period of 4 and 16 weeks. **(A)** Spleen weight. **(B)** IFNγ, and **(C)** IL-12p40 secretion of MLN cells isolated from SIHUMI or SIHUMI without *E. faecalis* colonized mice (colonization period: 4 weeks) that were re-activated with the respective bacterial lysate for 72 hours. **(D)** Correlation between *E. faecalis* abundance (luminal colon and cecum content) and mean histological scores of cecum tip and distal colon. **(E)** Serum IgA against SIHUMI species: Western blots of the indicated bacterial lysates using sera from SIHUMI or SIHUMI - *E. fae* colonized IL-10$^{-/-}$ mice. Differences were considered significant for *p≤0.05, ****p≤0.0001.

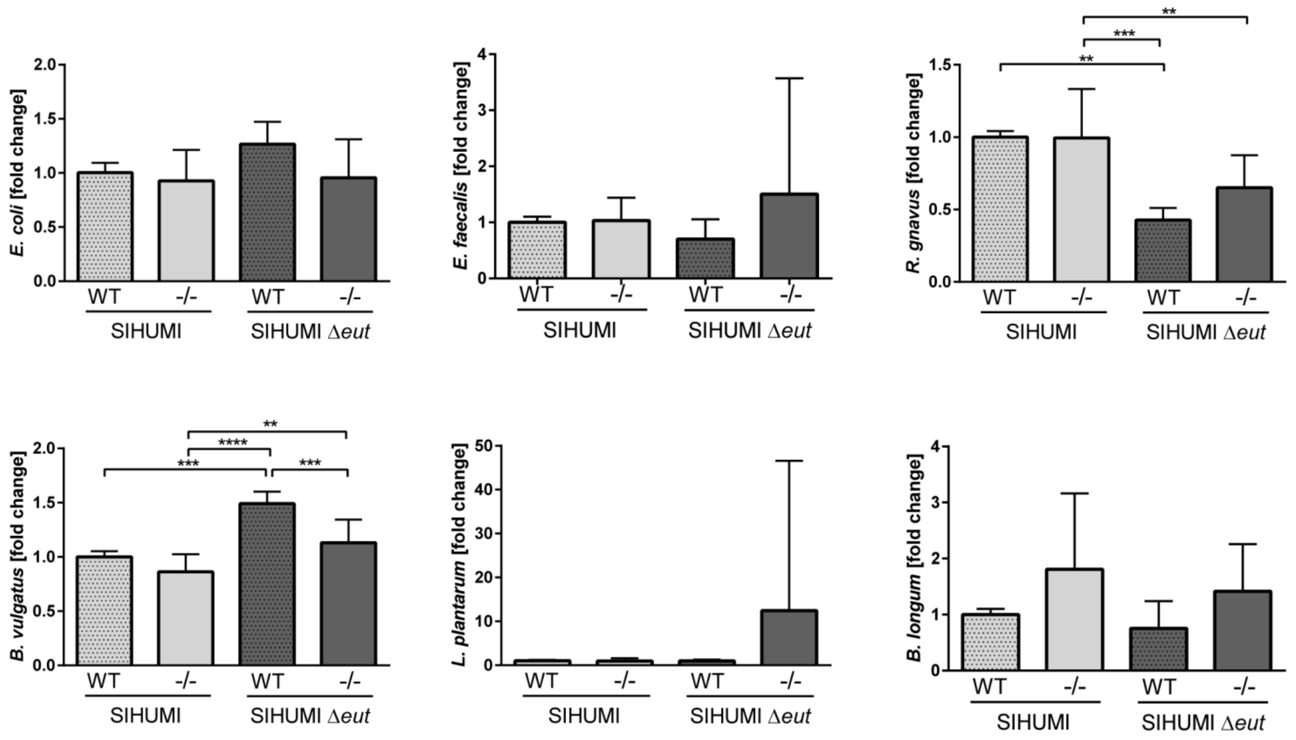

Supplementary Figure 5. Germfree WT and IL-$10^{-/-}$ mice were colonized with SIHUMI consortium including *E. faecalis* OG1RF (SIHUMI) or the Δ*eut* mutant (SIHUMI Δ*eut*) for 16 weeks. Quantification of specific SIHUMI bacteria in colonic contents using 16S rRNA gene targeted qPCR. Copy number is normalized to total 16S copy number and fold change is relative to SIHUMI colonized WT mice. Differences were considered significant for **$p \leq 0.01$, ***$p \leq 0.001$, ****$p \leq 0.0001$.

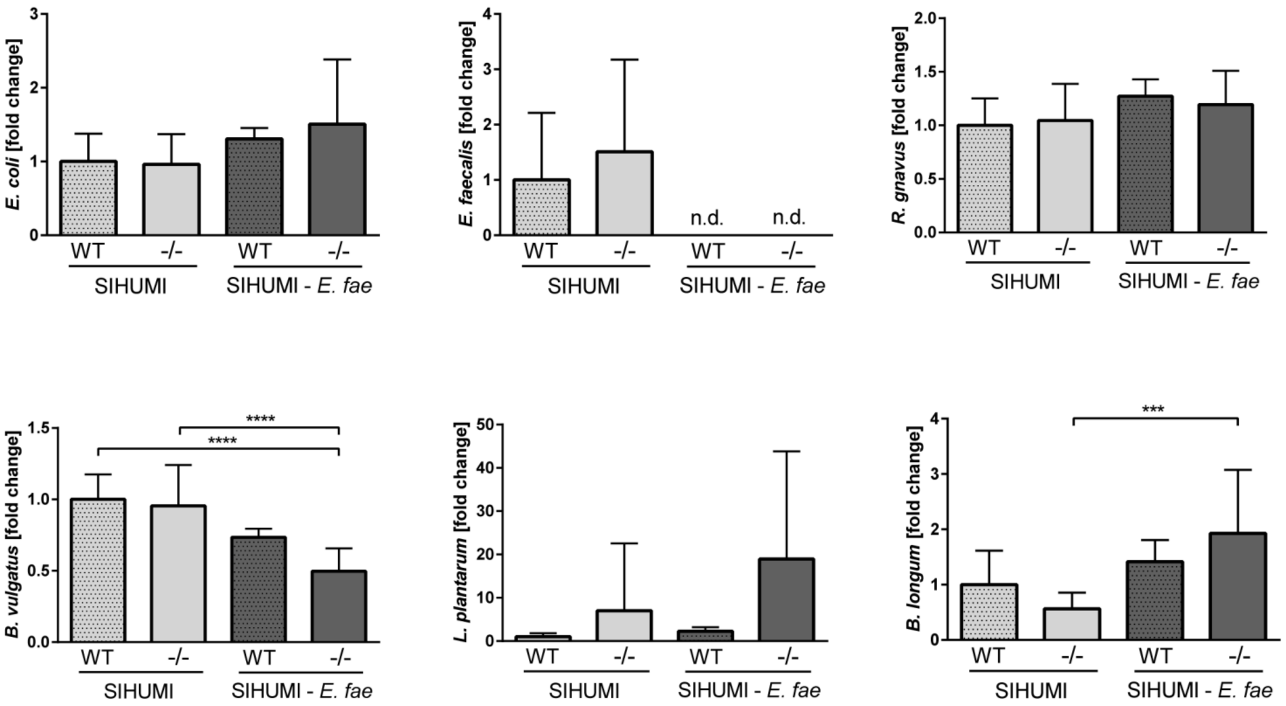

Supplementary Figure 6. Germfree IL-$10^{-/-}$ and wild type (WT) mice were colonized with SIHUMI consortium or SIHUMI consortium without *E. faecalis* (SIHUMI - *E. fae*) for 16 weeks. Quantification of specific SIHUMI bacteria in colonic contents using 16S rRNA gene targeted qPCR. Copy number is normalized to total 16S copy number and fold change is relative to SIHUMI colonized WT mice. Differences were considered significant for ***$p \leq 0.001$, ****$p \leq 0.0001$.

2 Supplementary Tables

Supplementary Table 1. Differentially regulated genes between *E. faecalis* isolated from monoassociated IL-$10^{-/-}$ mice versus wild type mice. Base mean: mean normalized counts, averaged over all samples from both conditions; log2(FC): the logarithm (to basis 2) of the fold change; StdErr: standard error estimate for the log2 fold change estimate; Wald-Stats: Wald statistic; P-value: p value for the statistical significance of this change; P-adj: p value adjusted for multiple testing with the Benjamini-Hochberg procedure which controls false discovery rate (FDR).

Supplementary Table 2. Differentially regulated genes between *E. faecalis* isolated from SIHUMI colonized IL-$10^{-/-}$ mice versus wild type mice. Base mean: mean normalized counts, averaged over all samples from both conditions; log2(FC): the logarithm (to basis 2) of the fold change; StdErr: standard error estimate for the log2 fold change estimate; Wald-Stats: Wald statistic; P-value: p value for the statistical significance of this change; P-adj: p value adjusted for multiple testing with the Benjamini-Hochberg procedure which controls false discovery rate (FDR).

3 Supplementary Methods

3.1 Animal experiments - Experimental design

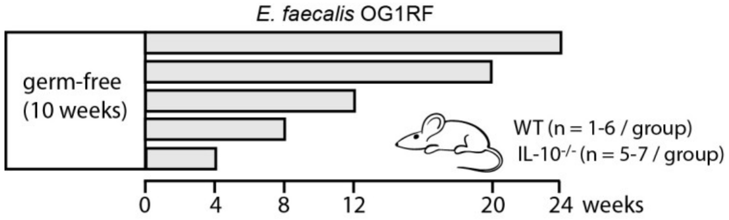

Supplementary Figure 7. Experimental setup: Wild type and IL-$10^{-/-}$ mice were monoassociated with *E. faecalis* for 4, 8, 12 and 24 weeks, respectively.

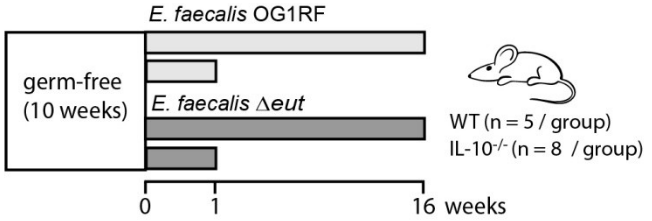

Supplementary Figure 8. Experimental setup: Wild type and IL-$10^{-/-}$ mice were monoassociated with *E. faecalis* wild type (OG1RF) or the Δ*eut* mutant for 1 and 16 weeks, respectively.

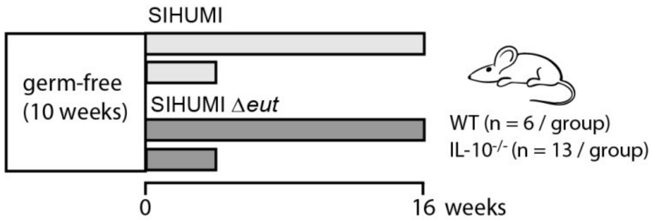

Supplementary Figure 9. Experimental setup: Wild type and IL-$10^{-/-}$ mice were colonized with SIHUMI consortium including *E. faecalis* wild type (SIHUMI) or the Δ*eut* mutant (SIHUMI Δ*eut*) for 16 weeks.

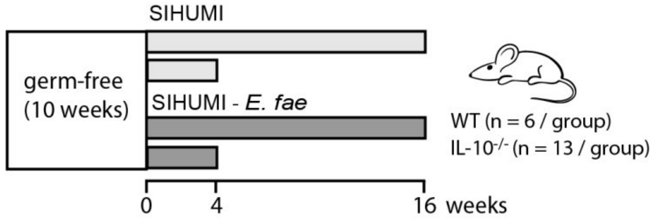

Supplementary Figure 10. Experimental setup: Wild type and IL-$10^{-/-}$ mice were colonized with SIHUMI consortium or SIHUMI consortium without *E. faecalis* (SIHUMI - *E. fae*) for 4 and 16 weeks, respectively.

3.2 Droplet Digital PCR (ddPCR, Bio-rad)

DNA isolated from pure bacterial culture or colonic content of germfree or SIHUMI colonized mice was digested with BamHI-HF (New England Biolabs) for 1 h. The restriction digest was used in a final concentration of 50 ng in the ddPCR mixture containing 10 µl of 2x ddPCR Supermix for Probes (Bio-Rad), 900 nM of forward and reverse primers and 125 nM of the respective UPL probe (Roche) in a total reaction volume of 20 µl. The primers and probes are shown in Table 3. The 20 µl reaction mixture and 70 µl per reaction of Droplet Generation Oil for Probes were loaded into a droplet cartridge. After droplet generation, the emulsion was transferred into a 96 well plate and PCR was performed in a C-1000 thermal cycler (Bio-Rad) with following conditions: 95°C for 10 min, 40 cycles of 94°C for 30 s and 60°C for 1 min. PCR-positive and PCR-negative droplets were detected using a droplet reader (Bio-Rad). The Quantasoft software was used for downstream analysis.

3.3 SDS-PAGE and immunoblotting (IgA immunoreactivity analysis)

Bacterial lysates were mixed with TruPAGE 4x LDS buffer (Sigma Aldrich) and denaturated for 10 min at 70°C. The lysates were subjected in a concentration of 3 µg/lane to discontinuous denaturing polyacrylamid gel electrophoresis (SDS-PAGE), transferred onto a polyvinylidene difluoride membrane (PVDF, GE Healthcare Amersham) and blocked at RT for 1 h in 5% skim milk-TBST (0.05% Tween in TBS). After overnight incubation with a 1:500 dilution of mouse serum (pool of serum from six wild type or ten IL-$10^{-/-}$ mice) in 5% skim milk-TBST, the membrane was washed 3 times in TBST, incubated with a 1:1000 dilution of goat anti mouse immunoglobulin A (IgA) FITC conjugate (Invitrogen) and washed 3 times with TBST. Antibody binding was detected using a Typhoon Trio+ imager (GE Healthcare Amersham).

3.4 Ethanolamine quantitation in colonic content by LC-MS/MS analysis

Quantitation of ethanolamine in colonic content from wild type and $IL10^{-/-}$ mice monoassociated with *E. faecalis* OG1RF or Δ*eutVW* was performed at the Bavarian Center for Biomolecular Mass Spectrometry (BayBioMS). Sample preparation and mass spectrometry quantitation was modified after Bertin et al. (2011). Briefly, colonic content was diluted 1:20 with purified ddH_2O (Millipore) and debris was removed by two centrifugation steps at 4°C (2000×*g* for 20 min, 6000×*g* for 5 min). One ml of colonic content was spiked with 65 µl of a 1 mg/ml solution of ethanol-1,1,2,2-d_4-amine (Sigma Aldrich) as internal standard. For protein removal, 1 ml supernatant was incubated with 100 µl 40% sulfosalicylic acid (Sigma Aldrich), followed by centrifugation at 11000×*g* for 15 min. Precipitated proteins were removed and ethanolamine (EA) was derivatized with dansyl chloride (Sigma Aldrich) for mass spectrometry quantitation. For derivatization, supernatants were mixed with 300 µl of 0.5 M $NaHCO_3$ (Merck), 2 ml of 10 mg/ml dansyl chloride in acetone (Sigma Aldrich)

and 200 µl of 1 M NaOH (Sigma Aldrich). The samples were incubated for 20 min at RT in the dark and residual dansyl chloride was removed by addition of 200 µl 25% NH_4OH (Sigma Aldrich). Subsequently, the sample volume was adjusted to 5 ml with acetonitrile (Sigma Aldrich) and 10 µl of the solution was injected for LC-MS/MS analysis. The LC-MS/MS system consisted of a Shimadzu-Prominence LC system, including a LC-20AD pump, a DGU-20A3 degasser, a SIL-20A autosampler and a CTO-20A column oven, with a QTRAP 4000 mass spectrometer equipped with an Turbo V ion source (Applied Biosystems). Analyst software (Applied Biosystems, version 1.6.2) was used for instrument control, data acquisition and data processing. Chromatographic separation was performed on a Kinetex 5u XB-C18 100 A column (100 x 2.1 mm, 5µm, Phenomenex) using water/formic acid (100/0.1 *v/v*) as solvent A and acetonitrile/formic acid (100/0.1, *v/v*) as solvent B at a flow rate of 400 µl/min and a column temperature of 40°C. Starting with 5% solvent B, the amount of B in the mobile phase was increased to 100% in 3 min and hold for 2 min, followed by re-equilibration at starting conditions for 3 min. The mass spectrometer was operated at 450°C in the positive electrospray ionization mode with the ion spray voltage set at 5500 V. Dansyl-ethanolamine was detected with MRM mode using the following settings: *m*/*z* 295.1/157 (entrance potential (EP) 10, declustering potential (DP) 70, collision energy (CE) 37, cell exit potential (CXP) 7, quantifier), 295.1/170 (EP 10, DP 72, CE 31, CXP 8, qualifier) and dansylethanol-1,1,2,2-d_4-amine was used as internal standard:*m*/*z* 299.1/158 (EP10, DP 70, CE 37, CXP 7).

3.5 Preparation of bacterial lysates

Bacterial lysates were prepared as described previously (Eun et al., 2014). Shortly, individual bacterial colonies were inoculated in 20 ml of broth for 24 h. The bacterial cultures were washed twice with and resuspended in sterile PBS. Per 1 ml bacterial suspension, 250 µl freshly prepared MD solution (0.1 M $MgCl_2$, 100 µg/ml DNase I) was added. Bacteria were disrupted by using a FastPrep-24 (MP Biomedicals) under addition of 0.1-mm glass beads and the suspension was sterilized using a 0.2 µm syringe filter.

3.6 Bacterial RNA isolation

Bacterial total RNA was isolated as described previously (Ridaura et al., 2013). Briefly, bacterial cells were mechanically disrupted using glass beads of 0.1 mm in a FastPrep-24 bead beater (MP Biomedicals). RNA was isolated by phenol:chloroform:isoamyl alcohol (25:24:1, pH 4.5, Carl Roth) extraction, precipitated with isopropanol and purified using the NucleoSpin RNA extraction kit (Macherey-Nagel). RNA concentration and purity was analyzed using a NanoDrop 1000 spectrophotometer (Thermo Fisher Scientific). The RNA integrity was assessed by Gel Electrophoresis. Contaminating genomic DNA was digested using TURBO DNase (Thermo Fisher Scientific) according to the manufacturer's instructions.
